# Multi-dose Romidepsin Reactivates Replication Competent SIV in Post-antiretroviral Rhesus Macaque Controllers

**DOI:** 10.1371/journal.ppat.1005879

**Published:** 2016-09-15

**Authors:** Benjamin B. Policicchio, Cuiling Xu, Egidio Brocca-Cofano, Kevin D. Raehtz, Tianyu He, Dongzhu Ma, Hui Li, Ranjit Sivanandham, George S. Haret-Richter, Tammy Dunsmore, Anita Trichel, John W. Mellors, Beatrice H. Hahn, George M. Shaw, Ruy M. Ribeiro, Ivona Pandrea, Cristian Apetrei

**Affiliations:** 1 Center for Vaccine Research, University of Pittsburgh, Pittsburgh, Pennsylvania, United States of America; 2 Department of Infectious Diseases and Microbiology, Graduate School of Public Health, University of Pittsburgh, Pittsburgh, Pennsylvania, United States of America; 3 Department of Pathology, School of Medicine, University of Pittsburgh, Pittsburgh, Pennsylvania, United States of America; 4 Department of Microbiology and Molecular Genetics, School of Medicine, University of Pittsburgh, Pittsburgh, Pennsylvania, United States of America; 5 Department of Medicine, Perelman School of Medicine, University of Pennsylvania, Philadelphia, Pennsylvania, United States of America; 6 Division of Laboratory Animal Resources, School of Medicine, University of Pittsburgh, Pittsburgh, Pennsylvania, United States of America; 7 Department of Medicine, School of Medicine, University of Pittsburgh, Pittsburgh, Pennsylvania, United States of America; 8 Theoretical Biology and Biophysics Group, Los Alamos National Laboratory, Los Alamos, New Mexico, United States of America; Emory University, UNITED STATES

## Abstract

Viruses that persist despite seemingly effective antiretroviral treatment (ART) and can reinitiate infection if treatment is stopped preclude definitive treatment of HIV-1 infected individuals, requiring lifelong ART. Among strategies proposed for targeting these viral reservoirs, the premise of the “shock and kill” strategy is to induce expression of latent proviruses [for example with histone deacetylase inhibitors (HDACis)] resulting in elimination of the affected cells through viral cytolysis or immune clearance mechanisms. Yet, *ex vivo* studies reported that HDACis have variable efficacy for reactivating latent proviruses, and hinder immune functions. We developed a nonhuman primate model of post-treatment control of SIV through early and prolonged administration of ART and performed *in vivo* reactivation experiments in controller RMs, evaluating the ability of the HDACi romidepsin (RMD) to reactivate SIV and the impact of RMD treatment on SIV-specific T cell responses. Ten RMs were IV-infected with a SIVsmmFTq transmitted-founder infectious molecular clone. Four RMs received conventional ART for >9 months, starting from 65 days post-infection. SIVsmmFTq plasma viremia was robustly controlled to <10 SIV RNA copies/mL with ART, without viral blips. At ART cessation, initial rebound viremia to ~10^6^ copies/mL was followed by a decline to < 10 copies/mL, suggesting effective immune control. Three post-treatment controller RMs received three doses of RMD every 35–50 days, followed by *in vivo* experimental depletion of CD8^+^ cells using monoclonal antibody M-T807R1. RMD was well-tolerated and resulted in a rapid and massive surge in T cell activation, as well as significant virus rebounds (~10^4^ copies/ml) peaking at 5–12 days post-treatment. CD8^+^ cell depletion resulted in a more robust viral rebound (10^7^ copies/ml) that was controlled upon CD8^+^ T cell recovery. Our results show that RMD can reactivate SIV *in vivo* in the setting of post-ART viral control. Comparison of the patterns of virus rebound after RMD administration and CD8^+^ cell depletion suggested that RMD impact on T cells is only transient and does not irreversibly alter the ability of SIV-specific T cells to control the reactivated virus.

## Author Summary

Antiretroviral therapy (ART) does not eradicate HIV-1 in infected individuals due to virus persistence in latently infected reservoir cells, despite apparently effective ART. The persistent virus and can rekindle infection when ART is interrupted. The goal of the “shock and kill” viral clearance strategy is to induce expression of latent proviruses and eliminate the infected cells through viral cytolysis or immune clearance mechanisms. Latency reversing agents (LRAs) tested to date have been reported to have variable effects, both on virus reactivation and on immune functions. We performed *in vivo* reactivation experiments in SIV-infected RMs that controlled viral replication after a period of ART to evaluate the ability of the histone deacetylase inhibitor romidepsin (RMD) to reactivate SIV and its impact on SIV-specific immune responses. Our results suggest that RMD treatment can increase virus expression in this setting, and that it does not markedly or durably impair the ability of SIV-specific T cells to control viral replication.

## Introduction

Viral reservoirs are infected cells that persist even in the face of seemingly effective suppressive antiretroviral therapy (ART) and can give rise to recrudescent infection when ART is stopped. Reservoir cells include latently infected resting, memory CD4^+^ T cells, as well as other cells, such as T memory stem cells (TSCM) or T follicular helper cells (Tfh) [1–8]. Cells harboring latent proviruses carry the virus for the duration of their lifespan. As the half-life of central memory T helper cells is estimated at 44 months [9], and even longer for the TSCM and Tfh [6,10,11], and latently infected cells that do not express viral antigens are invisible to immune clearance mechanisms, such cells can persist for decades, even in patients successfully treated with ART [12–16]. Upon stochastic reactivation, perhaps in connection with homeostatic proliferation or antigen specific stimulation, these quiescent cells can revert their status and start producing new virions [5,17]. Even if expression of viral antigens results in immune clearance, the virus will persist as long as proliferation equals or exceeds clearance. ART may suppress most *de novo* infections of susceptible cells by virions derived from reactivated cells, but viral rebound occurs after variable delays at the cessation of ART, with plasma viral load (PVLs) typically rebounding to pretreatment levels [18,19]. With an estimated 1 latently infected cell per 1x10^6^ CD4^+^ T cells [20], current paradigms predict that the latent viral reservoir is unlikely to be naturally eliminated over the lifetime of an HIV-infected individual on ART [21,22].

With the reports of the Berlin patient, the Boston patients and the Mississippi baby, there has been renewed interest in the prospect of achieving viral eradication, or at least sufficient reduction of the reservoir to allow extended viral remission in the absence of continuous ART, both considered as acceptable “HIV cure” strategies [23–26]. Current cure approaches include ART intensification studies [27–31], infusion of CCR5-gene modified CD4^+^ T cells following chemotherapy [24,32], enhancement of host HIV-specific immune responses to remove reactivated cells, and variations on the “shock and kill” approach [33–40]. The “shock and kill” strategy has seen the most emphasis. This approach consists of the administration of latency reversing agents (LRAs) to induce expression of latent proviruses, with the cells in which virus expression is induced being eliminated by viral cytopathic effects or host immune responses. *De novo* infection of susceptible cells by LRA-induced virus is prevented by ongoing ART [41,42]. This strategy represents a logical approach that theoretically should eventually result in the curbing/elimination of the reservoir. Several types of LRAs have been tested and have shown at least a limited success in activating the latent reservoir, including histone deacetylase inhibitors (HDACis) [38,43,44], protein kinase C (PKC) agonists, such as bryostatin-1 and prostratin [35,45] and the bromodomain-containing protein 4 inhibitor JQ1 [34,36]. Of these, HDACis have shown the most promise in reactivating the virus from the reservoirs. Valproic acid, givinostat, entinostat, vorinostat (suberoylanilide hydroxamic acid, SAHA), panobinostat and romidepsin (RMD) are the most studied HDACis; their effects on reactivating the virus from the reservoir are variable [38,42,46–48]. RMD has been shown to be among the most potent inducers of HIV in both *in vitro* and *ex vivo* models [38]. Recent studies have also documented modest activity of RMD to increase plasma viremia in both HIV-infected patients [49] and SIV-infected macaques [50] on ART. However, RMD has also been reported *in vitro* to inhibit host CTLs that target infected cells [51].

Elimination of reactivated resting CD4^+^ T cells in which expression of latent proviruses is induced by LRAs is proving to be more challenging than originally proposed [52]. Initially, it was believed that LRAs capable of potent and extensive induction of latent proviruses could be identified and that upon reactivation, infected cells would be killed by cytopathic effect (CPE) and/or host immune responses. Though logical, experience to date has not fulfilled these expectations. Specifically, studies have demonstrated that: only a small fraction of proviruses present in resting CD4^+^ T cells are reactivated by a single round of HDACi treatment *in vitro* [53]; reactivation of infected, resting CD4^+^ T cells by vorinostat did not result in viral CPE-mediated cell death [42]; impairment of host HIV-specific CTLs’ functions is specifically associated with immunodeficiency characteristic to HIV infection even in treated patients [54]; viral clearance by the CTLs only occurs in elite controllers [55]; the effect of various LRAs on latently infected cells, both *in vivo* and *ex vivo*, is highly variable [45,56,57]; *in vitro* treatment of cells with HDACi can impair CTL function [51]; and, finally, much of the virus present in latently infected cells in individuals that started ART in the chronic phase of infection contains escape mutations for immunodominant epitopes [58].

Therefore, research is currently refocusing on improving the efficiency of viral induction, for example through combinatorial approaches with LRAs targeting different mechanisms that contribute to the establishment and maintenance of viral latency, and boosting the killing of cells expressing induced virus through enhancing host immune responses, particularly the CD8^+^ CTL responses, by way of therapeutic vaccines, monoclonal antibodies and immune checkpoint inhibitors [39,59–62]. Note, however, that complex combinatorial strategies, in which LRAs are combined with immunomodulators, may add difficulty to data interpretation (as was the case with the Berlin patient [63]).

In this context, to evaluate different strategies for targeting viral reservoirs, animal models are needed that reproduce key features of HIV infection, while representing experimentally tractable systems that allow a mechanistic evaluation of different interventions. In addition to permitting testing of strategies for which lack of proof of concept or safety concerns may preclude clinical evaluation, animal models, particularly NHP models, offer the opportunity for extensive tissue sampling, to assess what is, after all, a tissue-based disease [64,65]. Yet, current animal models have their own limitations: (i) For years, SIVmac infection in RMs was more difficult to control than HIV-1 infection, requiring the use of complex and expensive ART regimens [50,66–69], and only recently, a simplified coformulated regimen was reported to effectively control SIVmac infection in RMs [70,71]; (ii) Humanized mice models, though suitable for addressing some questions relevant to cure research [65,72–74], do not allow detailed assessment of potential virus reservoirs due to limitations on samples obtainable from individual animals and on the feasible durations of treatment.

The “Visconti cohort” is a group of French HIV-infected individuals in which prolonged ART was initiated early in infection but eventually halted, and in which a fraction of patients managed to control virus rebound in the absence of continued ART, despite the lack of protective MHC alleles or other known factors that might lead to such an outcome [75]. We developed a NHP model to replicate post-ART control of viral replication in RMs infected with an infectious molecular clone (SIVsmmFTq). This model permits characterization of both the host factors associated with post-treatment control of viral replication and of the dynamics of the viral reservoir in post-treatment controllers. Furthermore, our model can be used to test virus reactivation strategies in the presence of apparently effective immune responses (in a “shock and effective kill” approach). Thus, our model permits the study of LRA efficacy on viral induction without confounding factors such as ART and immunotherapy. We used this model to assess the ability of the HDACi RMD to reactivate virus. We report that RMD can effectively increase virus expression in this model of post-treatment viral control and that RMD administration did not induce a marked or durable alteration of the cellular immune responses *in vivo*.

## Results

### Generation of a RM model of post-treatment viral control after ART

**Fig 1:**
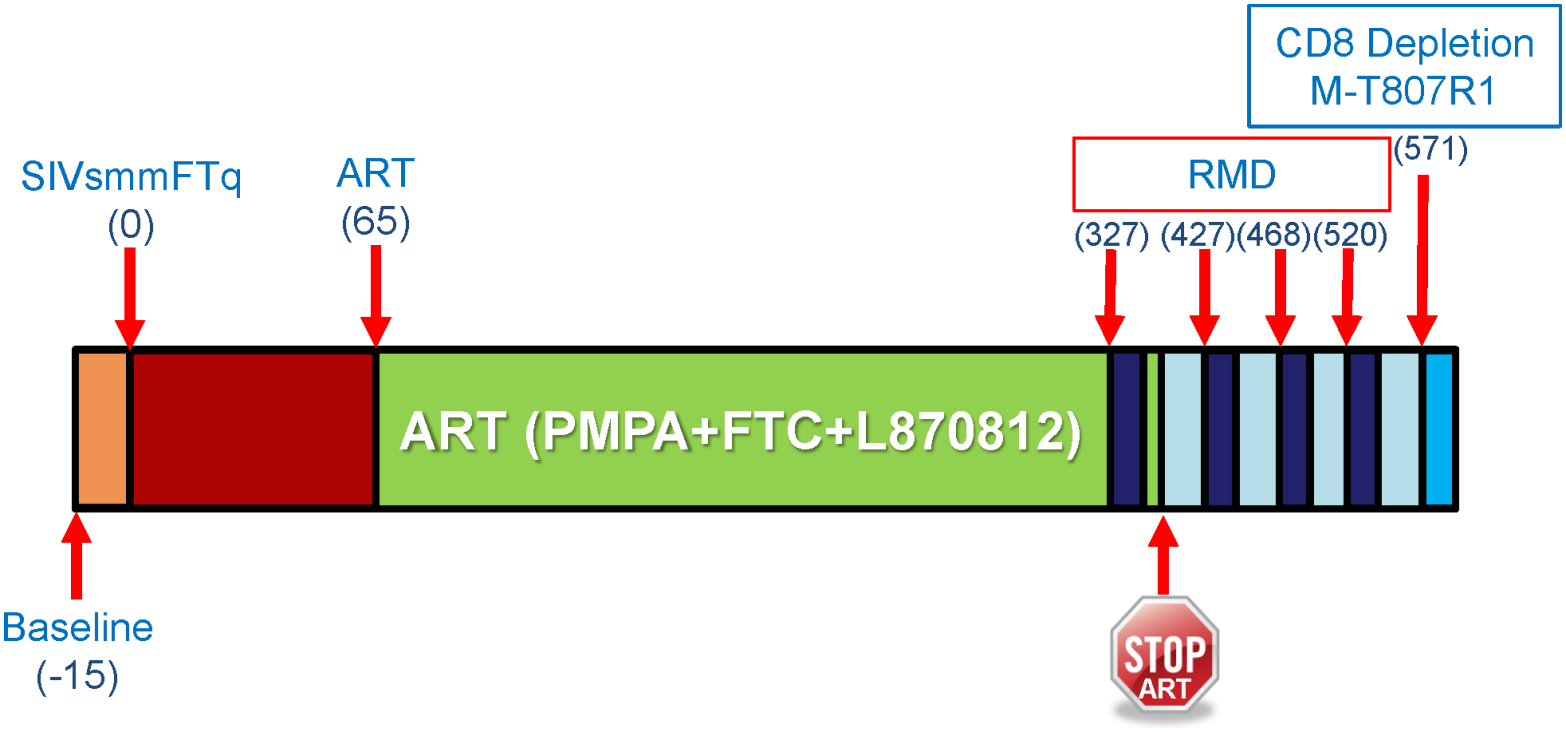
Study design. The timeline of the study indicates the days in which SIVsmmFTq infection was performed, ART was initiated, RMD was administered, as well as the experimental time point of the CD8^+^ cell depletion with M-T807R1 mAb. Sampling time points are not shown, as only blood was collected in this study and the time points of blood collection are too numerous to be shown. Please refer to the method section for information regarding the sampling schedule.

To establish a macaque model of post-ART control of virus for studies of approaches to target viral reservoirs that persist in this setting, we identified a SIVsmm strain (FTq) [76] that replicates in RMs at levels that are similar to those observed in chronically HIV-1-infected patients. We developed a transmitted/founder (TF) infectious molecular clone (IMC) of SIVsmmFTq, using the methodology reported in Gnanadurai *et al*. [77] and used this new IMC to intravenously infect ten Indian RMs. Six RMs were used as controls, in which SIVsmmFTq infection followed its natural course in the absence of any therapeutic intervention, while the remaining RMs served as a study group and received ART, followed by virus reactivation with RMD, as illustrated in Fig 1.

**Fig 2:**
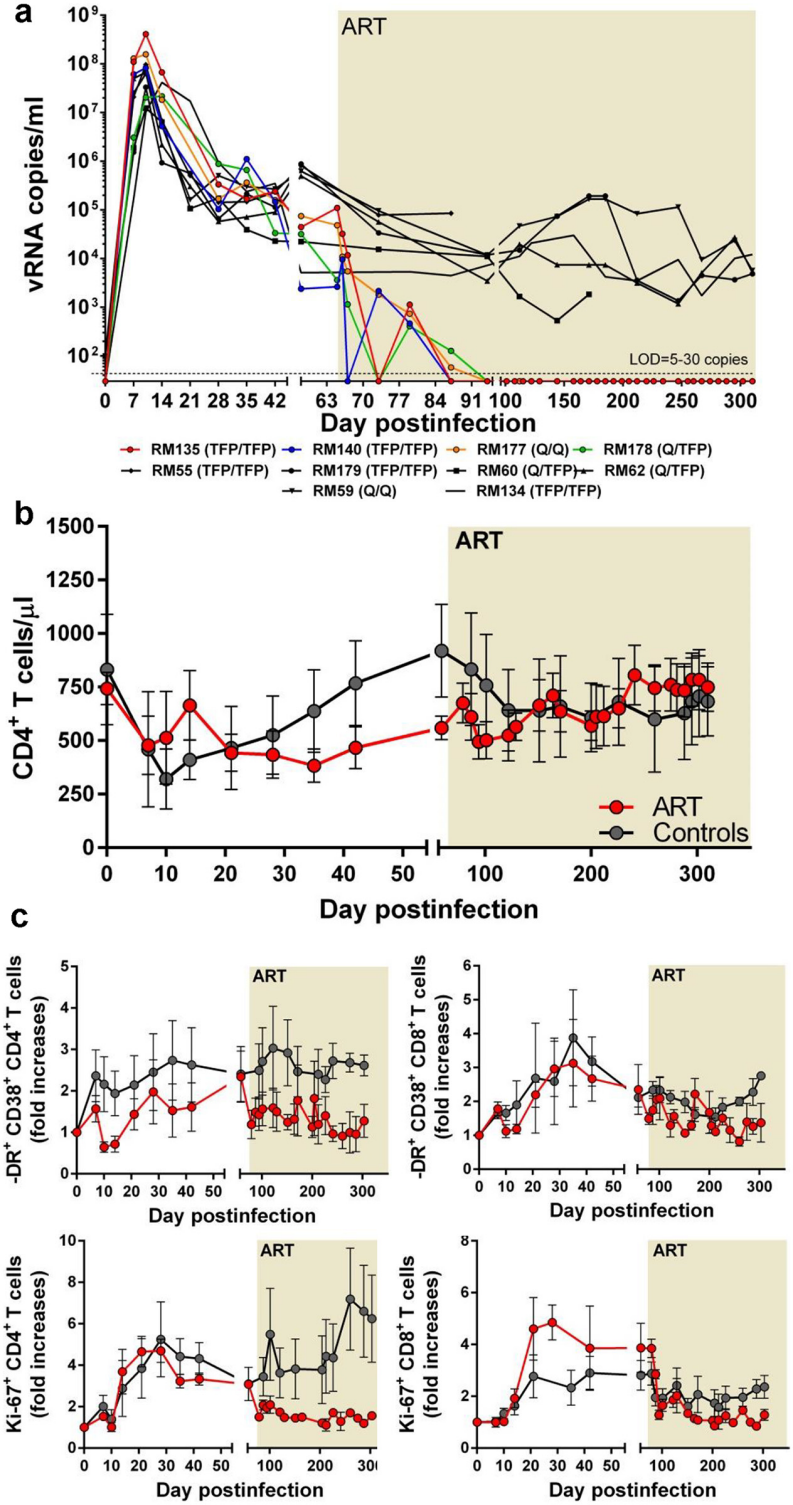
SIVsmmFTq infection of RMs can be completely controlled with conventional antiretroviral therapy (ART). (a) Comparative dynamics of viral replication between RMs in the control group (black symbols and lines) and the RMs receiving a combination ART (PMPA + FTC + L-870812) for a duration of >9 months (treated animals are color-coded, as shown in the legend). (b) Comparison of the changes in peripheral CD4^+^ T cells between naïve RM controls (black lines and symbols) and RMs receiving ART (red lines and symbols). (c) Comparison of the changes in the frequency of immune activation of peripheral CD4^+^ and CD8^+^ T cells between naïve RM controls (black lines and symbols) and RMs receiving ART (red lines and symbols). Duration of ART highlighted in the graphs. The limit of detection (LOD) for the viral load assay is depicted (a) and ranged between 5 and 30 copies/ml depending on the sample availability. In Figs b and c, the lines represent the average values for the monkeys in the groups, and the bars represent the standard errors of the means (sem).

During >1 year follow-up, we show that SIVsmmFTq closely reproduced the patterns of virus replication observed in HIV-1 infection, with high PVL peaks [10^7^−10^8^ viral RNA (vRNA) copies/mL] and a robust, but relatively controlled replication during chronic infection (10^4^−10^5^ copies/mL) (Fig 2a). Furthermore, this robust virus replication resulted in a significant depletion of peripheral CD4^+^ T cells during the acute SIVsmmFTq infection and a partial CD4^+^ T cell restoration during chronic infection (Fig 2b).

The set-point levels of chronic SIVsmmFTq replication being in the range of HIV-1 infection, and lower than those observed with SIVmac239 [78], we reasoned that, similar to HIV-1, SIVsmmFTq can be readily controlled with ART. Four RMs intravenously infected with SIVsmmFTq received a combination of tenofovir (PMPA), emtricitabine (FTC) and integrase inhibitor (L-870812) for over nine months, starting at 65 days postinfection (dpi). ART resulted in a multiphased decay of plasma viremia with PVLs decreasing to <10 copies/ml in all RMs receiving ART by 30 days on ART, at 95 dpi (Fig 2a). During the follow-up, our priority was to assess the robustness of viral control and assess whether or not viral blips occurred under ART. To this end, RMs on ART were sampled every three days. This very frequent sampling schedule limited the amount of plasma available for viral quantification, preventing us from lowering the detection limit below 10 copies/mL. However, at this detection limit, no detectable vRNA blips were recorded in the plasma over the following 8 months of treatment in any of the RMs receiving ART, confirming our hypothesis that the administered ART regimen successfully controlled SIVsmmFTq replication in these RMs.

During treatment, one of the RMs on ART (RM177) died of unrelated conditions (complications of anesthesia), at 112 dpi (52 dpt). At the time of death, plasma viremia was < 10 copies/mL in this monkey (Fig 2a).

The robust control of viral replication in RMs receiving ART impacted the recovery of the CD4^+^ T cells, which, in spite of being similarly depleted from circulation in both groups of SIVsmmFTq-infected RMs during acute infection, recovered to nearly preinfection levels in the RMs treated with ART (9 months of ART, with 8 months of plasma viremia < 10 copies/mL). Comparatively, the control RMs showed a more limited restoration at the end of the follow-up (Fig 2b).

Furthermore, the fractions of CD4^+^ and CD8^+^ T cells expressing immune activation markers were lower in RMs receiving ART compared to controls (Fig 2c). Note, however, that, similar to HIV-infected patients on ART [79,80], and other models of ART-treated SIV-infected RMs [66], a low level of residual immune activation persisted during antiretroviral therapy in SIVsmmFTq-infected RMs, despite of a robust viral control with ART. These features of the SIVsmmFTq-infected RM model more closely recapitulate key aspects of HIV infection of humans compared to the highly pathogenic SIVmac239 infection.

### RMD administration to SIVsmmFTq-infected RMs on ART does not result in a detectable rebound of PVL

After demonstrating that conventional ART can robustly control SIVsmmFTq replication, we next attempted to reactivate the virus from the reservoir through administration of RMD. One dose of 7 mg/m^2^ of RMD was administered to the three RMs on ART in a slow perfusion over four hours. Blood samples were obtained during and after RMD treatment to assess the pharmacological effects of the RMD, as well as virus reactivation.

**Fig 3:**
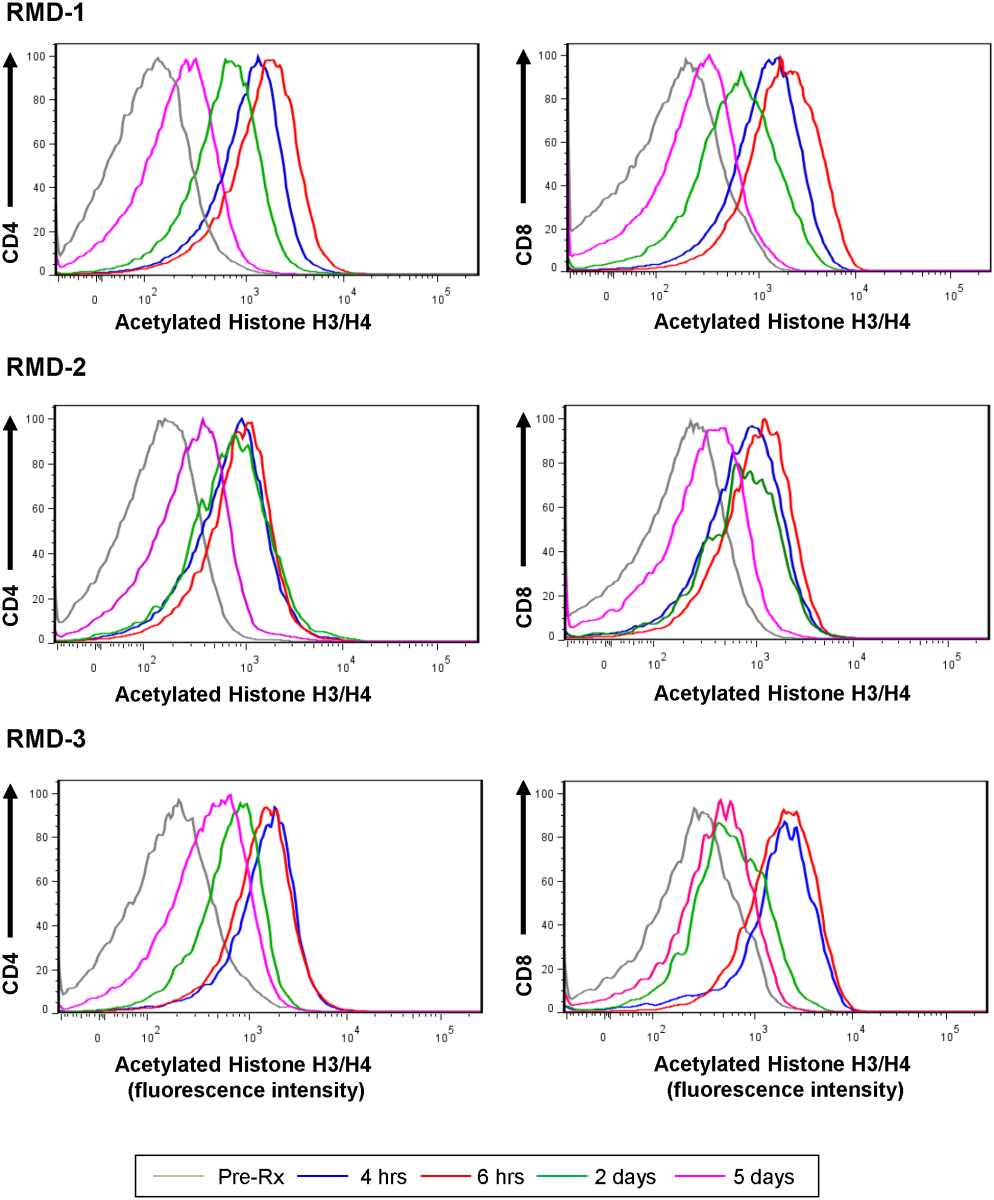
Romidepsin increases the levels of acetylated histones in RMs. The levels of acetylated histones have been measured by flow cytometry prior to RMD administration, and at 4 hrs, 6 hrs, 2 days and 5 days after RMD administration. Testing was performed after the first, the second and the third RMD administration. Only the results from RM135 were shown, but data were representative for all animals receiving RMD.

**Fig 4:**
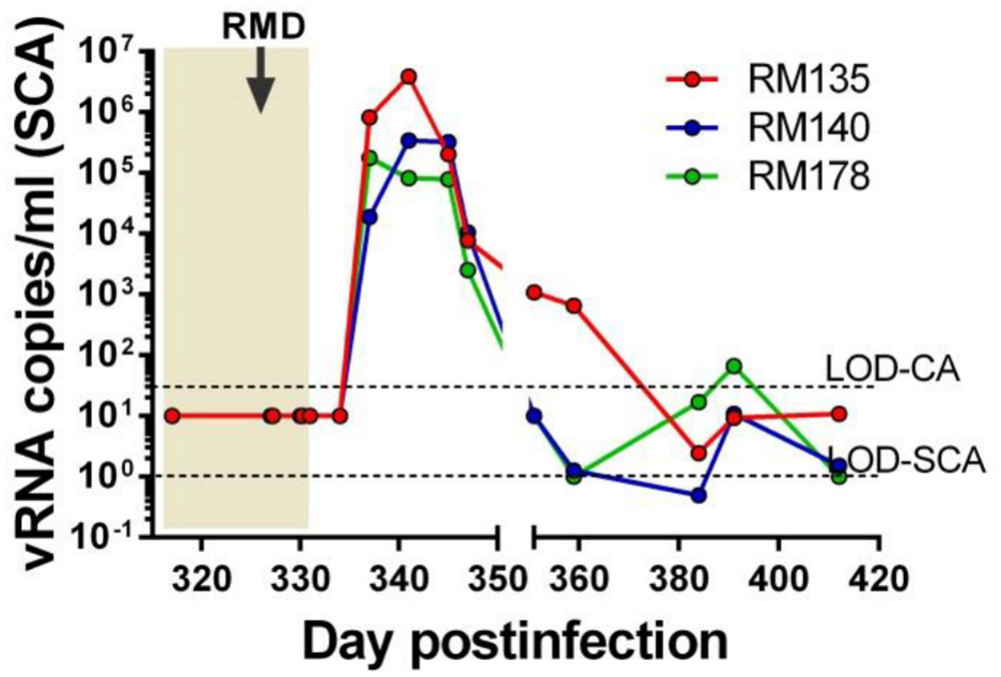
PVLs prior to and after RMD administration in RMs on antiretroviral therapy (ART) and virus rebound at the cessation of ART. Longitudinal PVLs were measured with a conventional assay (CA), with a limit of detection (LOD) of 30 viral RNA (vRNA) copies/ml of plasma (the top dashed line). PVLs were also measured with an ultrasensitive single copy assay (SCA), with a limit of detection of 1 vRNA copy/ml of plasma (bottom dashed line). During the follow-up, based on sample availability, the sensitivity of SCA ranged between 5–10 viral RNA copies/ml. Time of the RMD administration is illustrated with a black arrow.

We first monitored RMD activity by measuring acetylated histone (H3 and H4) levels in both CD4^+^ and CD8^+^ T cells [66]. Histone acetylation increased during the RMD treatment peaked at 6 hours post-RMD treatment initiation and returned to nearly pretreatment levels by 5 days post treatment (dpt), confirming that we had delivered a bioactive dose of the drug (Fig 3). However, in spite of the documented increase of the levels of acetylated histones, we did not observe any measurable increase in plasma viremia after RMD administration to RMs on ART (Fig 4).

Therefore, one week after completion of RMD treatment, ART was stopped in all RMs. Cessation of ART was followed by rapid and robust rebound of plasma viremia in all three RMs. Viral rebound: (i) occurred very rapidly, with SIVsmmFTq being detected in plasma only three days after ART cessation (Fig 4); (ii) was higher than expected, reaching peak levels of 10^5^−10^7^ copies/ml (Fig 4), higher than the set-point PVL established during chronic infection, which is often the level of virus rebound observed at the cessation of ART [81]; and (iii) was controlled to < 30 copies/ml (below the limit of detection of our conventional assay) within 50 days after discontinuation of ART (Fig 4). We continued to closely monitor PVLs in these RMs using a more sensitive assay and observed that PVLs fluctuated between ≤10 copies/ml and 30 copies/ml, but no animal lost control of the virus over 150 days of observation. Based on the characteristics of the post-treatment dynamics of viremia, these three RMs were labelled as post-treatment controllers [75] (Fig 4).

### Multi-dose RMD reactivates replication-competent SIV in post-treatment controllers *in vivo*

Due to the unexpected characteristics of the PVL rebound leading to eventual post-treatment control of infection, we designed a new strategy to test whether or not the transient “excess of viral rebound” that followed ART interruption can be attributed to RMD administration. To assess the ability of RMD to induce viral expression in the setting of post-ART spontaneous viral control, we administered three doses of RMD at 35–50 day intervals to the three post-treatment controller RMs.

After each treatment, RMD had detectable *in vivo* activity, as illustrated by increased levels of acetylated histones which peaked at 6 hours postadministration, and returned to nearly pretreatment levels by 5 dpt, as illustrated in Fig 3. Notably, repeated RMD administration did not result in changes in the levels of acetylated histones, which would have suggested tolerance. As this clear impact on histone acetylation was consistently observed after each RMD administration, we next assessed the ability of RMD to induce increased viral expression.

**Fig 5:**
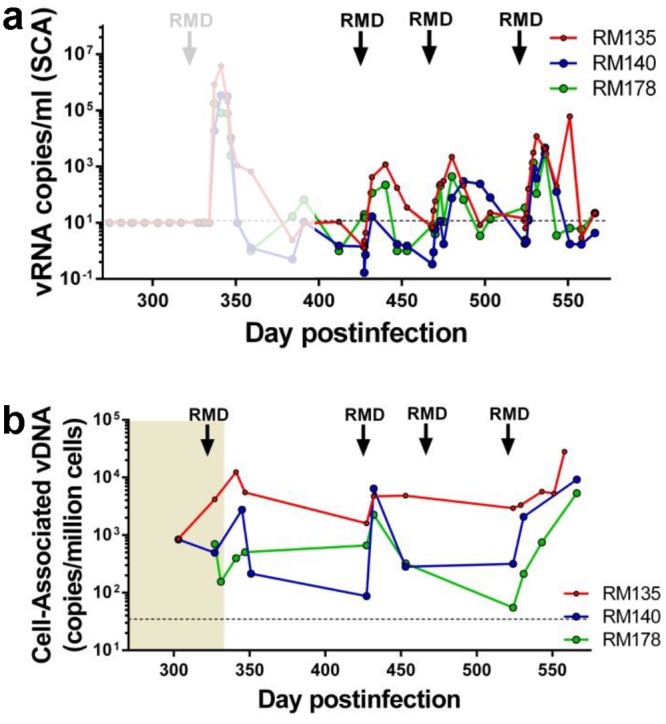
SIVsmmFTq reactivation after RMD administration in post-treatment controller RMs, as monitored by measuring the levels of PVLs and total CD4^+^ memory T cell-associated vDNA. (a) PVLs were measured using SCA, with a LOD of 1 copy/ml. Due to limitations in sample availability, the actual sensitivity of the SCA was of 5–10 copies (illustrated as a dashed line at 10 copies/ml). For comparison, the dynamics of PVLs at the cessation of ART are shown in shadow. (b) Total vDNA levels measured in memory CD4^+^ T cells from circulation. Times of the RMD administration are indicated with black arrows.

Virus reactivation was monitored by measuring the levels of vRNA in plasma with a single copy PCR assay (SCA) specifically developed for SIVsmmFTQ, similar to other previously described assays [82,83]. While no detectable rebound of PVLs could be documented in the plasma samples collected at 4, 6, 24 and 48 hours post-RMD administration, detectable PVLs were observed starting from 5 dpt in the RMD-treated RMs. Rebounding PVLs peaked at up to 10^4^ vRNA/ml by 13 dpt. After each RMD administration, this consistent virus rebound was gradually controlled to less than 10 copies/ml by 34 dpt (Fig 5a). With repeated RMD administrations, we observed increasingly robust virus rebounds, as documented by increased PVL peaks and longer delays to virus control (Fig 5a). We concluded that RMD has the ability to reactivate the controlled virus *in vivo* in post-treatment controller RMs. The delays in control of virus rebound, as well as their relative robustness may be due to the fact that, in the absence of ART, new cycles of replication occurred, permitting viral detection in plasma. As such, our study design allowed us to both confirm the efficacy of RMD in reversing latency and document that the virus reactivated after RMD administration is replication-competent. Yet, our results also point to the key observation that the levels of reactivated virus seen with RMD in the presence of ongoing ART are relatively low (as PVLs were below the SCA limit of detection immediately after RMD administration) and suggest that only amplification by *de novo* rounds of infection in the absence of ART allowed us to observe the effect.

In the absence of ART, RMD administration did not have a significant impact on the levels of total vDNA from CD4^+^ memory T cells. Thus, after each round of RMD administration, the levels of vDNA transiently increased.This was due to the study design, which allowed the virus to complete cycles of replication, resulting in the seeding of short-lived memory cells (i.e., effector memory cells). As these short-lived cells are productively infected and thus rapidly eliminated, the levels of vDNA in memory cells rapidly returned to pretreatment levels between RMD administrations (Fig 5b).

### Modeling the effect of RMD

To analyze the effect of RMD in more detail, we developed a simple dynamical model of virus production (see Methods), which shows that the slopes of increase of (the logarithm of) virus after each cycle of RMD in the absence of ART are related to the enhancement in viral production due to RMD. We estimated the slope of increase in PVLs using a linear-mixed effects model. We found that this slope was not significantly different across the three cycles of RMD treatment in the absence of ART, nor across RMs. The estimated slope was 0.418 log_10_/day (s.e. 0.037). The dynamical model indicates that the increase in viral production over baseline is proportional to this estimated slope (see Methods). Therefore, we can estimate that the increase in viral production due to RMD was between 1% and 5% of the baseline production before RMD, depending on how fast virus is cleared (100 day^-1^ or 20 day^-1^, respectively–see Methods for details). Assuming that at baseline production is in balance with viral clearance (P_0_ = cV_0_), these percentages allow us to estimate that the average increase in total body virus production attributable to RMD was only between ~150 and ~8000 virions per day.

### Multi-dose RMD does not induce major or lasting toxicity in SIV-infected RMs

Due to the nature of RMD administration by slow perfusion and the potential complications of prolonged anesthesia, all the animals treated with RMD received fluids throughout the time of drug administration. They also received Boost/Ensure *via* gavage at the last bleed on day of infusion, then again at 1, 2 and 3 dpt in order to compensate for the effects of prolonged sedation and extensive bleeding which could have reduced their appetite. In these conditions, we did not observe any major adverse effects of RMD in any RM, with the exception of a slight weight loss (probably due to frequent anesthesia), that recovered by 15 dpt. There were only minimal signs of toxicity after RMD administration, as suggested by the chemistry tests, which were normal at 5 dpt, with the exception of decreases in creatine kinase in all three animals and fluctuations in urea and total protein levels, as illustrated in S1 Fig. Similarly, complete blood counts (CBCs) did not show any major change in the blood cell populations indicative of drug toxicity (S2 Fig). Importantly, drug toxicity did not increase with repeated RMD administration. However, due to sample limitations, CBCs and chemistries were only performed prior and 5–7 days after RMD administration and these results should be treated with caution. Therefore, in an additional effort to assess potential RMD related toxicity, we closely monitored samples collected at multiple time points after each of the RMD treatments for levels of plasma lactate dehydrogenase (LDH), a marker of cell injury and death [84]. As illustrated in S3 Fig, RMD administration did not result in a significant increase in the levels of LDH in RMs (p = 0.459) (S3 Fig). These results suggest that RMD is effective and safe in RMs at the dose administered in our study.

**Fig 6:**
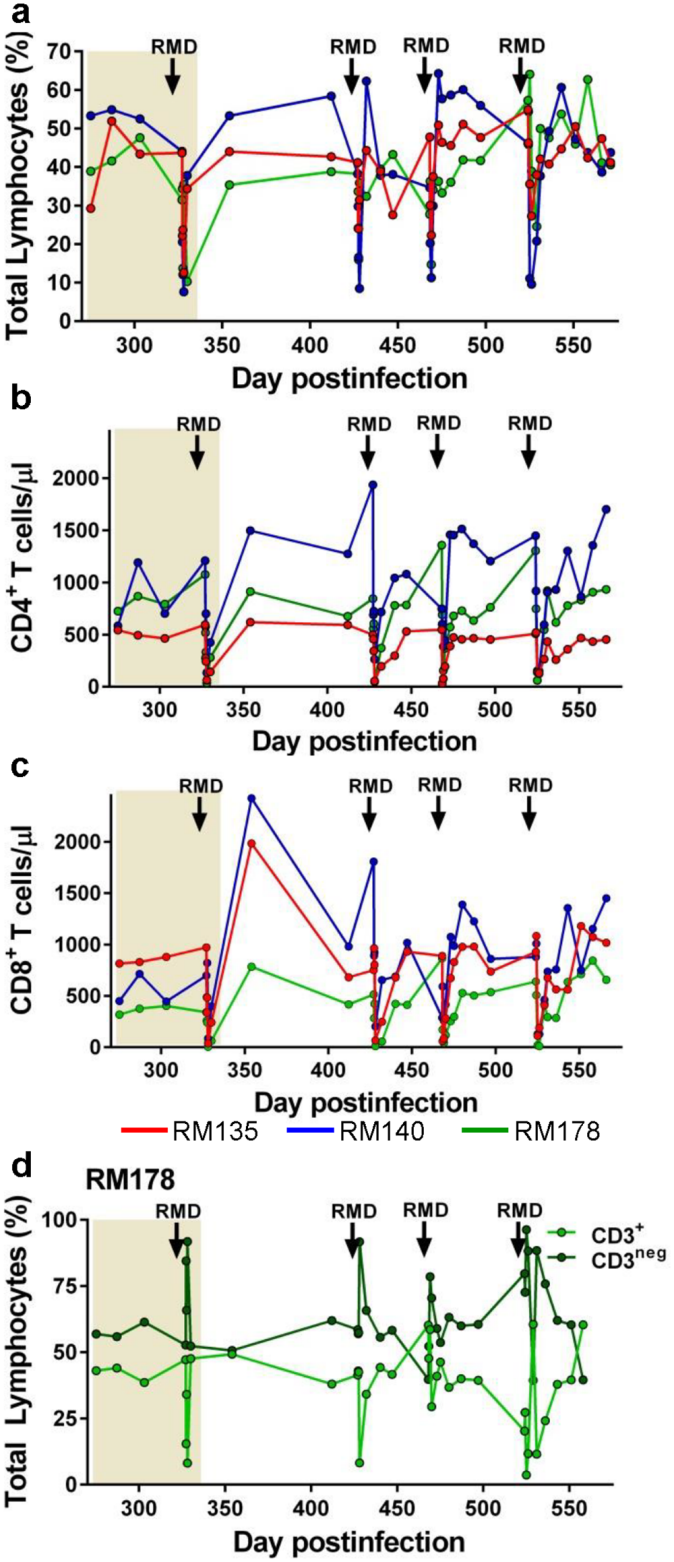
Effect of RMD administration on the peripheral blood major T cell subsets. (a) The levels of the total lymphocyte population (as measured by the Trucount assay) dramatically, but transiently, decrease after each round of RMD administration, resulting in similarly dramatic decreases of both CD4^+^ T cell (b) and CD8^+^ T cell (c) counts. (d) Comparative analysis of the CD3^+^ and CD3^neg^ populations showed that the reduction in the CD3^+^ T cell counts was mirrored by a dramatic surge in the population of CD3^neg^ lymphocytes, suggesting the significant reduction in the lymphocyte counts were due to a downregulation of surface markers rather than a real depletion of cells. ART administration is highlighted as a light orange box in the graphs. Time of the RMD administration is illustrated with black arrows.

However, in each of the treated RMs, after every RMD administration, a massive, but transient leukopenia was observed, with the overall levels of lymphocytes being reduced by an average of 76% (range: 55–86%) (Fig 6). Leukopenia occurred within 24 hours after RMD administration and lasted less than three days, with the lymphocyte levels being very rapidly restored to pretreatment levels within 5 dpt (Fig 6a and S2 Fig). This pattern was observed in all three RMs, and after every RMD administration, yet the total lymphocyte populations dramatically fluctuated in RM178 with more limited variations in the remaining two RMs (Fig 6a).

As a result, both CD4^+^ and CD8^+^ T cell counts were drastically reduced upon RMD administration (Fig 6b and 6c, respectively), but similar to the overall lymphopenia, they rebounded to pretreatment levels within one week (Fig 6b and 6c). Since T cell recovery after depletion is typically much slower, this rapid rebound suggests that the apparent reduction in T cell counts observed after RMD administration is not due to real cell depletion. We therefore monitored the CD3 expression on the surface of gated lymphocytes and identified a significant downregulation of CD3 following RMD administration (Fig 6d). The frequency of the CD3^+^ T cells in the lymphocyte gate decreased after RMD administration, with a concomitant increase in the frequency of CD3-negative cells (Fig 6d). As such, our results suggest that the apparent lymphopenia observed after RMD administration is due to downregulation of lymphocyte surface markers rather than a direct depletion of cells due to drug toxicity.

### Impact of RMD administration on the T cell activation and proliferation status

**Fig 7:**
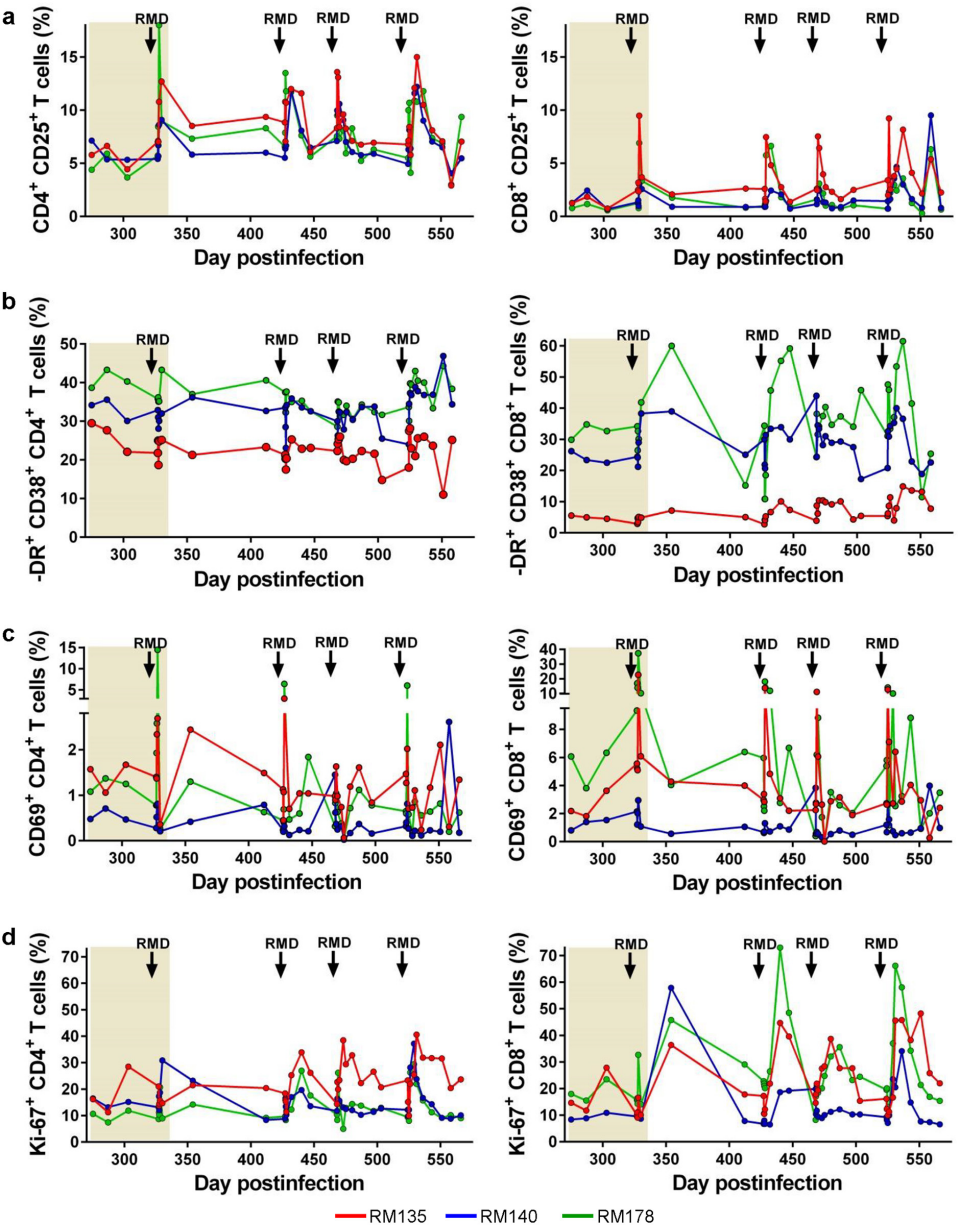
Multidose RMD administration exerts transient, but massive effects on the levels of T cell activation and proliferation. The fractions of CD4^+^ and CD8^+^ T cells expressing the immune activation markers (a) CD25, (b) HLA-DR and CD38, and (c) CD69 significantly increased after each RMD administration. (d) T cell proliferation (Ki-67) also increased after RMD administration. ART administration is highlighted as a light orange box in the graphs. Times of the RMD administration are illustrated with black arrows.

We next monitored levels of activation and proliferation after RMD administration by assessing the fraction of CD4^+^ and CD8^+^ T cells expressing the immune activation markers CD25 (Fig 7a), HLA-DR, CD38 (Fig 7b) and CD69 (Fig 7c), which increased only transiently in RMs treated with RMD. Increases in the levels of immune activation markers always preceded increases in PVLs suggesting that RMD can activate resting cells (S4 Fig).

The fraction of CD4^+^ and CD8^+^ T cells expressing the proliferation marker Ki-67 also increased significantly, but this increase tended to be slower than seen for CD69 [85] (Fig 7d). Thus, the fraction of CD4^+^ and CD8^+^ T cells expressing Ki-67 peaked at 12 dpt, paralleling viral replication (Fig 7d). The frequency of T cells expressing both Ki-67 and immune activation markers returned to pretreatment levels prior to subsequent RMD administration. Altogether, the dynamics of immune activation and proliferation markers, in combination with findings when the animals were administered RMD while on ART [50], suggested that these changes were due to RMD administration rather than a response to viral replication, at least in the initial stages after RMD administration.

### Romidepsin does not dramatically hinder SIV-specific T cell responses *in vivo*

A recent *ex vivo* study attributed the ability of RMD to reactivate HIV from the reservoir to a major effect exerted by this drug (similar to other HDACi) on immune cell effectors, through elimination of CD8^+^ T cells and a reduction of the cytolytic capabilities of CTLs [51]. As we also observed a major, albeit transient, lymphopenia in RMs after administration of RMD, we next assessed the impact of RMD on SIV-specific T cells *in vivo*.

**Fig 8:**
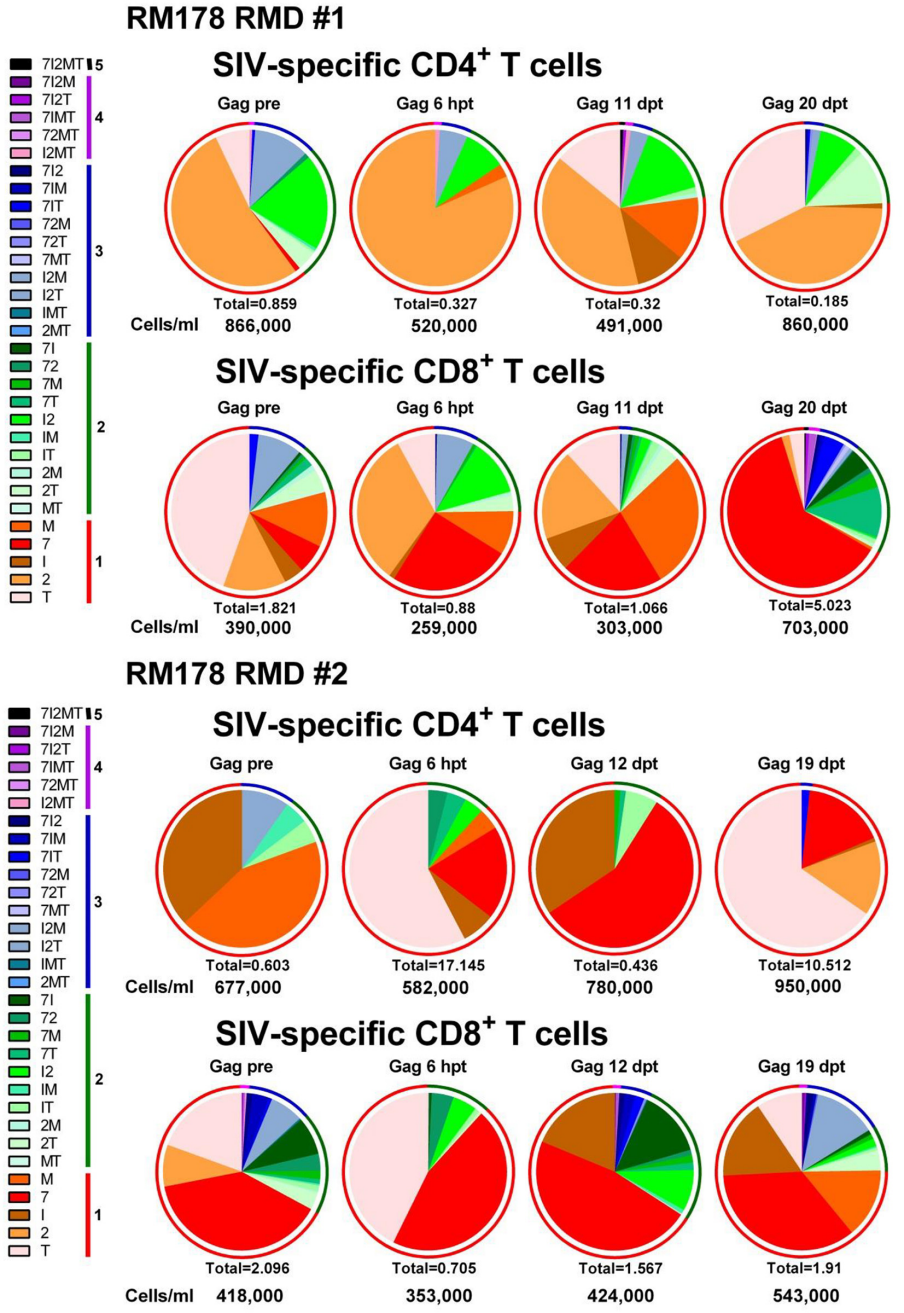
RMD administration does not significantly impact Gag SIV-specific T cell responses or functionality in SIVsmmFTq post-treatment controller RM178. Serial monitoring of SIV-specific T cell polyfunctionality after two rounds of RMD administration was achieved by stimulating PBMCs with Gag SIVmac239 peptide pools followed by intracellular cytokine staining. Cytokines tested include: TNF-α (T); IL-2 (2); IFN-γ (I); CD107α (7); and MIP-1β (M). Data are representative of all RMs. Absolute numbers of CD4^+^/CD8^+^ T cells/ml for each timepoint are presented beneath their respective pie graph. The pie charts depict functionality based on the combination of cytokines expressed, as illustrated in Fig legends. The color scheme represents the number of cytokines that were produced by the SIV-specific T cells (listed as numbers in the Fig legends) and the proportion of each is illustrated as a color-coded ring surrounding each pie chart to facilitate assessment of polyfunctionality.

**Fig 9:**
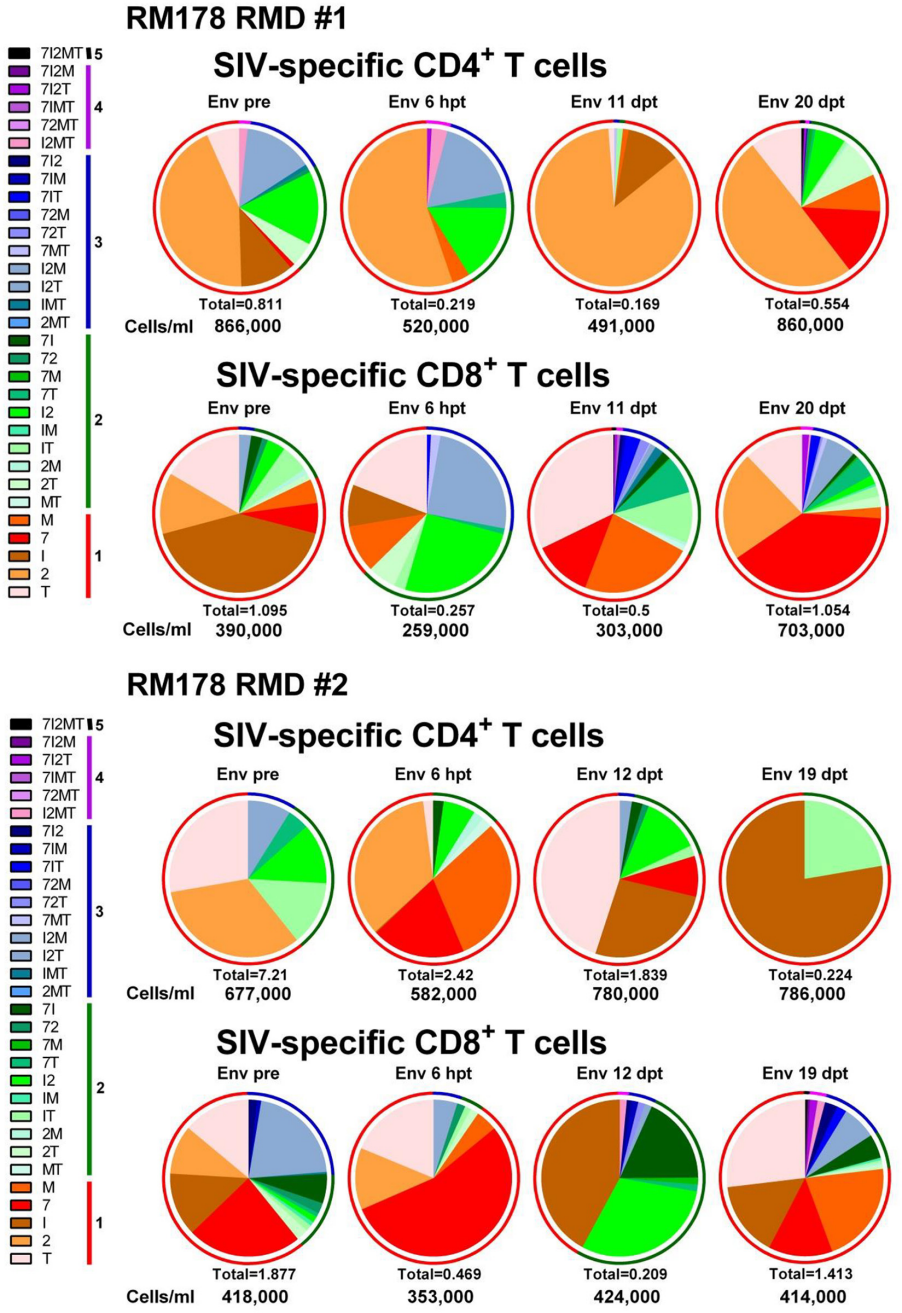
RMD administration does not significantly impact Env SIV-specific T cell responses or functionality in SIVsmmFTq post-treatment controller RM178. Serial monitoring of SIV-specific T cell polyfunctionality after two rounds of RMD administration was achieved by stimulating PBMCs with Env SIVmac239 peptide pools followed by intracellular cytokine staining. Cytokines tested include: TNF-α (T); IL-2 (2); IFN-γ (I); CD107α (7); and MIP-1β (M). Data are representative of all RMs. Absolute numbers of CD4^+^/CD8^+^ T cells/ml for each timepoint are presented beneath their respective pie graph. The pie charts depict functionality based on the combination of cytokines expressed, as illustrated in figure legends. The color scheme represents the number of cytokines that were produced by the SIV-specific T cells (listed as numbers in the figure legends) and the proportion of each is illustrated as a color-coded ring surrounding each pie chart to facilitate assessment of polyfunctionality.

**Fig 10:**
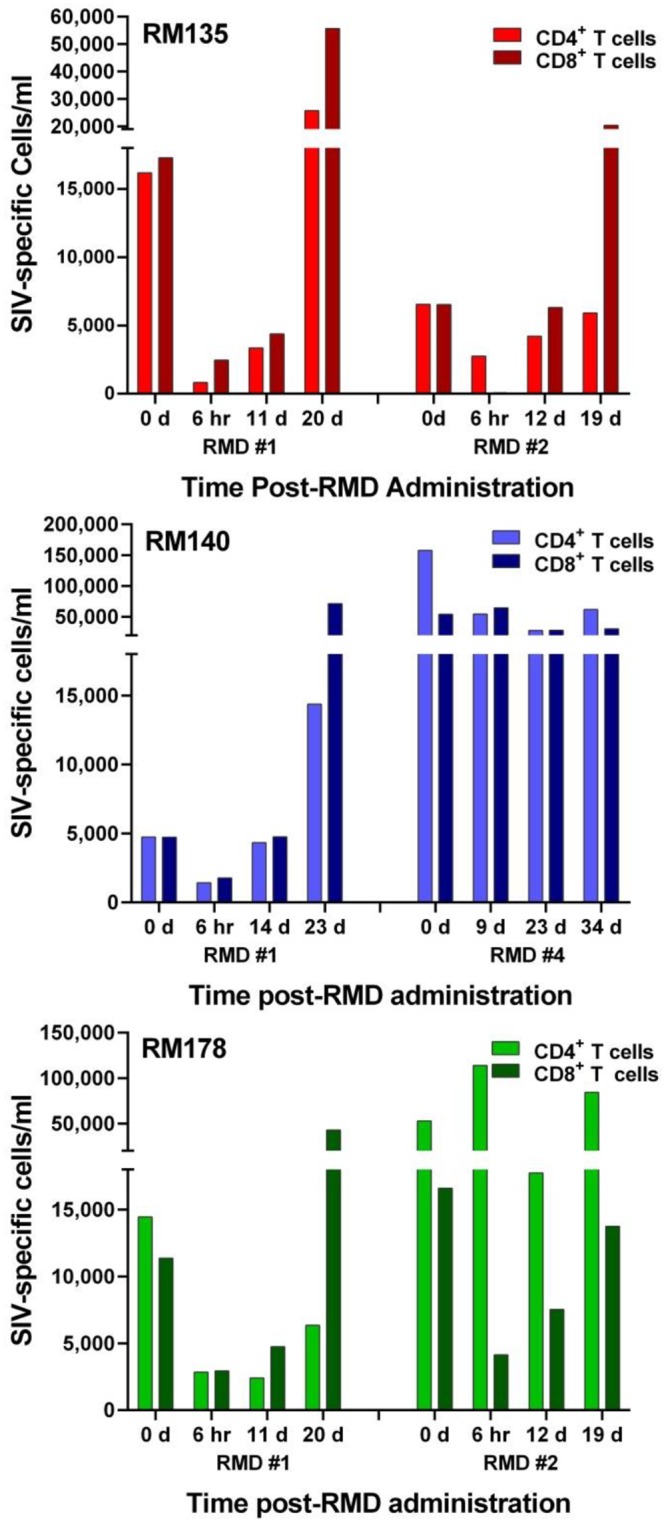
RMD administration does not significantly ablate the overall SIV-specific T cell responses in SIVsmmFTq post-treatment controller RMs. Absolute numbers (/ml) of the aggregated Gag- and Env-specific CD4^+^ and CD8^+^ T cells are shown at different time points following different rounds of RMD administration, as determined by intracellular cytokine staining and flow cytometry. On the X axis, the numbers represent days post-RMD administration.

Functional activity of both CD4^+^ and CD8^+^ T cells were monitored by intracellular cytokine staining (ICS) measurements of IL-2, TNF-α, IFN-γ, CD107α and MIP-1β production in response to stimulation with SIVmac239 Gag or Env peptide pools, measured in samples obtained various time points prior to and after RMD administration (Figs 8 and 9 and S5 and S6 Figs). ICS showed that RMD had only a transient impact on the absolute counts of Gag and Env-specific CD4^+^ and CD8^+^ T cells (Fig 10). Thus, while combined cytokine production was transiently hindered after RMD administration, SIV-specific T cell function was rapidly regained, before the viral control was reestablished (Figs 8 and 9 and S5 and S6 Figs). Furthermore, polyfunctionality of the CD4^+^ and CD8^+^ T cells was maintained or even boosted after RMD administration, probably as a result of the virus rebound representing a sufficient antigenic stimulus (Figs 8 and 9 and S5 and S6 Figs). The majority of the SIV-specific T cells were positive for the degranulation marker CD107α, which in ICS assays is considered a correlate of cytotoxic potential. Furthermore, the frequency of SIV-specific CD107α positive cells increased after RMD administration (Figs 8 and 9 and S5 and S6 Figs). The same pattern was observed after all RMD treatments. Together with the pattern of viral replication demonstrating control of the rebounding virus after each administration of RMD, our results suggest that in the post-treatment controller RMs that have functional immune responses, the virus reactivated through RMD administration can be effectively cleared by CTLs and that the impact of RMD on SIV-specific T cells is only transient and modest *in vivo*.

### Experimental ablation of CTL function through mAb mediated CD8^+^ cell depletion *in vivo*

**Fig 11:**
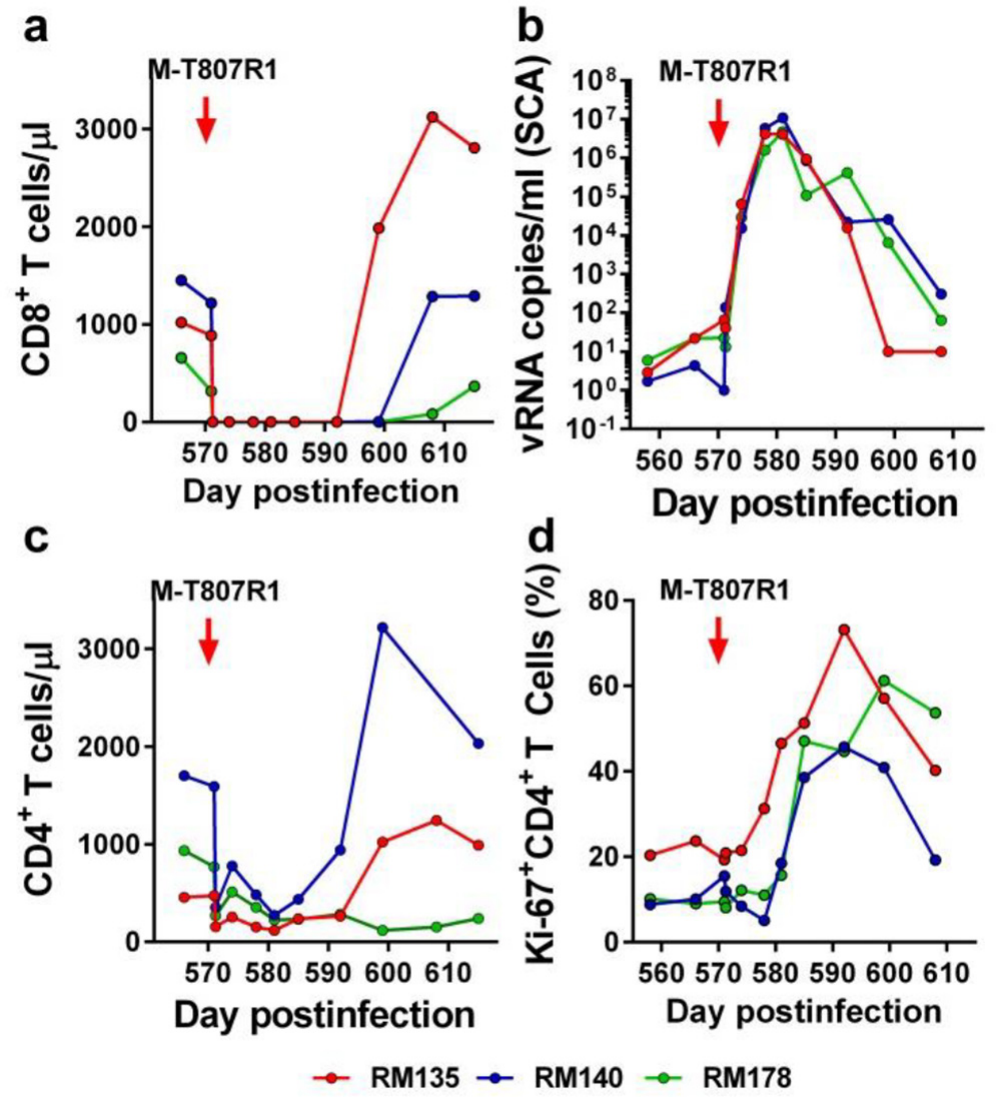
Experimental ablation of CTL responses through experimental CD8^+^ cell depletion (with the M-T807R1 anti-CD8 monoclonal antibody) result in patterns of viral rebound and immune activation that are different from those observed after RMD administration. Changes in the levels of (a) CD8^+^ T cells; (b) PVLs; (c) CD4^+^ T cells; and (d) CD4^+^ T cell proliferation (Ki-67) after administration of the M-T807R1 monoclonal antibody. Times of M-T07R1 administration are depicted as a red arrow.

Both the dynamics of the immune activation markers and their correlation with PVLs (Fig 7 and S4 Fig), as well as testing of the specific SIV responses, strongly suggested that virus rebound in the RMD-treated RMs was due to virus reactivation after LRA administration and not to loss of viral control through a major ablation of the CTL functions by HDACi. However, to further discriminate between the loss of control and virus reactivation, we modeled *in vivo* the ablation of CTL responses through direct experimental depletion of CD8^+^ cells. Post-treatment RM controllers received the M-T807R1 monoclonal antibody (mAb) to deplete CD8^+^ cells, after which plasma viremia, and the number and activation status of CD4^+^ T cells were compared and contrasted with the results observed after RMD administration. The anti-CD8 mAb successfully depleted peripheral CD8^+^ cells (Fig 11a) and loss of immune control was associated with a dramatic rebound of plasma viremia in all CD8-depleted RMs (Fig 11b). PVLs peaked at up to 10^7^ vRNA copies/ml by 10 days post M-T807R1 administration. As such, the PVLs observed after CD8^+^ cell depletion were orders of magnitude higher than those observed after RMD administration. PVLs were then slowly controlled over 5 weeks, much slower than after RMD administration, but mirroring the recovery of CD8^+^ T cells (Fig 11b). This massive viral replication resulted in a significant depletion of the CD4^+^ T cells in CD8^+^-depleted post-treatment controller RMs (Fig 11c). CD8^+^ cell depletion was also associated with a steady increase in the frequency of CD4^+^ T cells expressing Ki-67 (Fig 11d), which returned to predepletion levels after the rebound of CD8^+^ cells and control of PVLs.

When the levels of CD4^+^ T cell immune activation and proliferation markers were plotted on the PVLs in the CD8^+^-depleted post-treatment controller RMs, the viral rebound clearly preceded the increase in the levels of CD4^+^ T cell immune activation and proliferation markers (S7 Fig). This suggests that the observed virus rebound resulted from the ablation of the immune responses rather than from activation of the reservoir cells, as observed after RMD administration (S4 and S7 Figs).

Based on these results clearly documenting different patterns of viral rebound and control after RMD administration and CD8^+^ T cell depletion, we concluded that RMD administration does not trigger a permanent hindrance on CTL function and that virus rebound after RMD administration is due to the drug administration rather than to an ablation of CTL responses by RMD.

## Discussion

As research for a cure for HIV/AIDS gathers momentum, so does the use of animal models that can be employed to answer multiple key questions related to HIV infection pertinent to potential curative strategies, such as the location and structure of viral reservoirs, the impact of various therapeutic approaches on these reservoirs, as well as the toxicity of candidate LRAs [64]. These questions cannot be addressed without very invasive sampling and without major risks that can be achieved in animal models, but not in a clinical setting where the standard of care for HIV-infected individuals on ART means that in spite of being on chronic medication, they are otherwise able to have a virtually normal life, with a life expectancy that nears that of HIV-uninfected patients [86].

Here, we developed a model of post-treatment virus control of SIV infection that recapitulates features of the human post-treatment controllers [75]. RMs were intravenously infected with a new transmitted founder infectious molecular clone [77] derived from the strain SIVsmmFTq. This strain, which was identified during our previous surveys of SIVsmm diversity in Primate Centers in the US [76,87], has never been passaged *in vitro* and displays a lower pathogenicity in RMs than the highly adapted SIVmac/SIVsmm strains. We reasoned that since the set-point PVLs of this strain are lower than those of the reference SIVmac strains, SIVsmmFTq may be more readily controlled with ART. At 65 dpi, when the set-point viremia was achieved, but before major immune suppression occurred, RMs received an ART regimen consisting of the NRTIs PMPA and FTC and the integrase inhibitor L-812820, which is similar to the NRTI/Integrase inhibitor ART regimens containing Raltegravir or Dolutegravir used in combination of antiretrovirals recommended as first line therapy in HIV-infected patients [88]. ART was given continuously for over nine months, which we reasoned should ensure both completion of the first three stages of SIV decay [89,90], as well as a significant decay of the central memory T cells, the major component of the viral reservoir [91]. As shown by our results, our approach was effective, with PVLs controlled to <10 vRNA copies/ml for the duration of treatment, without any blips. Furthermore, at the end of treatment, the biological parameters improved in treated RMs compared to controls, with a trend to better preservation of CD4^+^ T cells and a partial control of T cell immune activation. Such profiles are characteristic to HIV-infected patients on ART [86,91].

We next assessed the usefulness of this new model for testing virus reactivation strategies. For these experiments, we chose RMD (94). The rationales for our choice were that HDACi are the most advanced class of LRAs, they are less toxic than other classes of LRAs (i.e., PKC agonists or the JQ1) [34–36,45], and that RMD is one of the most active HDACi [45,49,50]. SIVsmmFTq-infected RMs on ART received RMD at a dose of 7 mg/m^2^, two-fold higher than the dose previously used in RMs [50,92], but closer to the dose employed in human patients [93,94]. During and after treatment, we collected multiple samples and monitored both the effect of RMD on the levels of acetylated histones and the PVLs. We also monitored the side effects of the drug and report that these side effects were minimal, with the exception of dramatic, but transient lymphopenia, the mechanisms of which are currently being investigated in subsequent studies. The tight sampling schedule limited the amounts of plasma available for the SCA, and increased our limit of detection from 1 to 5–10 vRNA copies/ml. However, this is still a very high sensitivity and we could not detect any increase in PVLs after administration of RMD, in agreement with studies in HIV-infected patients, which suggested that RMD has only a limited effect on the reservoir [56]. Therefore, we decided to stop ART seven days after RMD administration.

A very rapid and massive virus rebound was observed upon ART interruption in all the SIVsmmFTq-infected RMs. Virus rebound was not unexpected, as in HIV-infected patients, rebound is nearly universal at the cessation of ART. However, detectable levels of SIVsmmFTq were quantifiable in plasma of RMs as early as three days after cessation of ART. This was more rapid than expected, considering that in patients in whom ART is initiated early during infection, similar to our RMs, the average time to detectable virus rebound is ~8 weeks [95,96], and is correlated with the duration on ART [96], the levels of HIV-1 DNA [97] and with the expression of T-cell exhaustion markers [98]. Even in patients starting ART during the chronic infection and in whom the immune system is exhausted, virus rebound occurs after an average of 2 weeks after ART interruption [99–101], longer than in the RMs in this present study.

Furthermore, while in HIV-infected patients the magnitude of virus rebound at the cessation of ART is generally similar to the set-point levels of viral replication prior to initiation of ART [81], in our study, the virus rebound was massive (up to 10^7^ vRNA copies), orders of magnitude higher than the set point PVLs established prior to treatment (i.e., 10^4^ vRNA copies/ml). Therefore, based on the characteristics of the virus rebound, we concluded that the excess of SIVsmmFTq replication observed at the cessation of ART was likely due to RMD, which had been administered only one week before. This was a first indication that RMD successfully contributed to virus reactivation.

Unexpectedly, in spite of its massive nature, likely to result in large-scale reseeding of the reservoir, the initial rebound was followed in all RMs by virus control below 50 copies/ml (the limit of detection of conventional assays). Such post-treatment control may raise questions relative to the relevance of our model, considering that ART interruption is associated with permanent loss of virus control in the vast majority of HIV-infected patients [99–101]. Note, however, that there is an important difference between the majority of HIV-infected patients, for whom ART is generally initiated during chronic infection, when they present with a significant immune suppression [99–101], and our RMs, in which ART was initiated during the initial stages of chronic infection. As such, our model of post-treatment control should be compared with HIV-infected patients in whom ART is initiated early and maintained for prolonged periods of time and for whom post-treatment control was reported to occur [75,96,102]. The most prominent case of post-treatment control is the Mississippi baby, in whom a very early initiation of ART (at 30 hours postdelivery) for a relatively long period of time (>18 months) resulted in post-treatment control and delayed virus rebound for 27 months [25]. Similarly, in the ANRS-Visconti cohort of patients, ART was initiated during acute infection for an average of 36 months and post-treatment control was reported to occur in 15% of subjects [75]. Moreover, while clinical trials using short course ART did not report post-treatment control, an impact on the reservoir size has been observed in these studies, resulting in a delayed virus rebound [95,96]. Finally, while a <6 month ART regimen initiated early in infection in SIVmac-infected RMs did not result in post-treatment control, a delay of virus rebound was associated with the early ART administration [71]. As such, the overall conclusion from these studies is that a sufficient reduction of the reservoir leading to a delayed virus rebound requires both early initiation and long duration of ART to allow the decay of central memory cells [60]. Here, we fulfilled both these requirements for the post-treatment control, with ART both initiated early in infection and maintained for duration roughly similar to the half-life of memory cells, and, as such, post-treatment control should not be completely unexpected. Also, note that in our newly developed moderately pathogenic SIVsmmFTq model we obtained a more robust control of viral replication with ART than during infection with the highly pathogenic SIVmac239, in which more aggressive ART regimens are frequently associated with less robust control of the virus and blips of viral replication [50,69,70].

In this context, it is tempting to speculate that the observed pattern of virus replication at the cessation of ART (i.e., massive rebound followed by control) is due to the fact that early and prolonged administration of ART before the total destruction of the immune system, together with the complete control of a moderately pathogenic virus, contributed to reservoir curbing. An alternative explanation is that preservation of effective cell-mediated immune responses (through a lower pathogenicity of the virus and an early initiation of ART) permitted effective control of the virus rebound. Future studies in animals on and off ART will allow us to detail the mechanism(s) of the post-treatment control in this model.

We next investigated whether or not RMD can reactivate the virus *in vivo*. We already had indications that RMD could have been at least partly responsible for the excess of viral replication compared to the pre-ART levels observed at treatment interruption. However, at the time of these experiments, the ability of HDACi to reactivate the latent virus and reduce the size of the reservoir was downplayed by *ex vivo* studies [45], as well as *in vivo* studies in humans and macaques on ART [66,103]. Therefore, it was critical to design a study which could unequivocally confirm the efficacy of RMD in reactivating the latent virus. There was also a debate in the field regarding the strategy of choice for measuring the reservoir. While authors were arguing that the inducible virus, which would be the main source of virus rebound in patients interrupting ART, can only be assessed by employing the quantitative virus outgrowth assay (Q-VOA) [104], other authors were arguing that Q-VOA underestimates the levels of inducible virus [105]. The major argument was that Q-VOAs were negative in both the Mississippi baby [26] and in the Boston patients [23], while the virus eventually rebounded in all these patients [23,25]. It was also argued that PCR-based methods overestimate the size of the reservoir and do not always correlate with the levels of inducible virus, as they detect defective viral genomes [106,107]. Since all these arguments were valid and no gold standard for the assessment of the viral reservoir is yet available, we reasoned that for our study, it would be best to use a conventional method for viral quantification to monitor the effects of RMD *in vivo* (i.e., PVL quantification). Such an approach would not only permit us to bypass controversies in the field, but would also allow us to simultaneously assess both the ability of RMD to reactivate the virus, and the replicative abilities of the reactivated virus, thus representing a valuable strategy to monitor LRA efficacy in preclinical *in vivo* screenings of new LRAs.

Our rationale was that without ART, the virus reactivated after RMD administration could reinfect new target cells, replicate and amplify, thus increasing the likelihood of detection with conventional PVL assays. We acknowledge that the main limitation of this strategy is that, in the absence of ART, the reactivated replicating virus will reseed the reservoir and prevent direct assessment of whether or not RMD reduces the reservoir. However, the most important question that we wanted to address was whether or not RMD can reactivate replication-competent virus from the reservoir and, thus, whether it has any future in cure research.

We performed three additional RMD administrations to RMs off ART, which resulted in an immediate and transient increase of T cell immune activation, returning to pretreatment levels within 24 hours. This T cell activation was followed by virus rebound in all the RMs treated with RMD. PVLs became detectable at 5 days post-RMD administration and peaked at 10^3^−10^4^ vRNA copies/ml by 13 dpt, thus demonstrating that RMD can indeed activate replication-competent virus from the reservoir. PVLs always followed the increases in the T cell immune activation levels, and therefore we concluded that virus rebound is most likely a result of the reservoir cell activation by RMD and not to loss of viral control through immune cell impairment after RMD administration. Meanwhile, we cannot discount the alternative explanation that the virus rebound might have resulted from the induction of an increased number of target cells, with the implication that RMD-induced SIV transcription may have occurred in only very few cells. In this second scenario, RMD impact on viremia might have been mostly indirect, through homeostatic effects.

To address these issues, using a simple model, we estimated that virus reactivation corresponded to between 1% and 5% of the pre-RMD viral production. Note that viral production may be underestimated, because we assumed that the drug has immediate effect, whereas there likely is a delay of at least a couple of hours post-treatment. Accounting for this delay would make the estimated slope of viral increase larger and hence production would also be larger. Our results suggesting a demonstrable, albeit limited, efficacy of RMD in reactivating the latent virus are supported by recent studies in both humans [49] and RMs [50].

To address an alternative explanation for the observed rebound, i.e., RMD-induced rapid suppression of cytokine production from viable T-cells and selective death of activated T cells, with the net result of impairing the activity of cytotoxic T-lymphocytes, as previously reported [51], we monitored the percentage of SIV-specific T cells in RMs after RMD administration. We report that, while documenting a reduction in the SIV-specific T cells immediately after RMD administration, we did not observe a substantial long-term impact of RMD on cell-mediated immune responses. However, we observed several interesting features that could help explain the results of the studies reporting the deleterious effects of RMD on CTLs. Immediately after RMD administration, we observed a massive, but very transient, reduction in the T cell counts, with the CD3^+^ T cell counts recovering in less than 5 days post-RMD administration. Due to the extremely transient nature of this reduction, we reasoned that it does not result from a real depletion of CD3^+^ T cells, but rather points to the downregulation of the surface markers used for cell counts. An alternative explanation for the observed kinetics of T cells might have been their redistribution to tissues, but in the absence of adequate tissue sampling, we could not assess this alternative. However, we showed that the CD3^+^ T cell reduction was mirrored by a dramatic increase of the negative cells in the lymphocyte gate. As such, it is possible that the effects of RMD on the cellular immune responses may be artefactual and are likely not the factor behind the observed virus rebound.

Finally, to understand whether or not the impact of RMD on cell-mediated immunity contributes to the observed results, we modeled the damage of cellular immunity through experimental depletion of CD8^+^ cells. RMs received the M-T807R1 mAb and the impact of CD8^+^ cell depletion was monitored by assessing both the levels of viral replication and those of immune activation. This experiment clearly showed that ablation of CD8^+^ cells contributed to a massive virus rebound, which was orders of magnitude higher than that observed after RMD. One may argue that CD8^+^ cell depletion is not comparable with the changes observed following RMD administration which occurs through a different mechanism and only partially impacts the CD4^+^ and CD8^+^ T cell populations. Further, the M-T807R1 monoclonal antibody impacts the NK cells, which may also contribute to the observed effects of CD8^+^ cell depletion. However, our major focus was on comparing the patterns of viral reactivation after RMD and CD8^+^ cell depletion. We report that, contrasting with RMD administration, in which virus reactivation was a result of increased T cell activation, virus rebound following CD8^+^ cell depletion preceded the increases in immune activation. Due to these clearly different patterns of virus rebound after RMD or CD8^+^ cell depletion, we concluded that the observed virus reactivation in our studies is due to RMD and not to impairment of CTL responses.

Our study design with RMD in the absence of ART, did not prevent the induced virus from reinfecting susceptible cells, leading to additional cycles of viral replication, reseeding the tissues, and altering the final size of the inducible viral reservoir. Further, due to sample size limitations, we could not perform a thorough characterization of the reservoir changes for the different rounds of RMD treatment. Thus, trying to assess in detail the impact of RMD on the reservoir would be an exercise in futility. However, quantification of the total memory cell-associated vDNA levels revealed that, while the size of the viral reservoir apparently did not significantly change between RMD administrations, a transient increase in the levels of memory cell-associated vDNA occurred after each RMD treatment. This might explain the trend to higher levels of viral reactivation after each additional round of RMD: virus seeding of the short-lived effector memory cell population leads to alterations in the composition of the viral reservoir. For example, we may speculate that, due to virus control during prolonged ART, in the initial viral RMD-induced reactivation, the rebounding virus likely originated nearly exclusively from long-lived resting central memory CD4^+^ T cells. However, with every round, the reactivated virus infects mostly susceptible cells (i.e., activated CD4^+^ memory cells) which could contribute to plasma virus in the subsequent rounds.

In conclusion, our results demonstrate that RMD may be successfully used to reactivate the latent virus and one may expect that, in the presence of an effective immune response, this intervention may curb the reservoir. Studies of virus reactivation in a background of ART, which are currently ongoing, will enable us to assess whether or not RMD administration can significantly reduce the size of the reservoir. Since the drug efficacy in reactivating the virus is rather modest, combination between different LRA classes or with immune modulators might be a strategy of choice for our attempts to reduce/eliminate the reservoir.

## Materials and Methods

### Ethics statement

All animals were housed and maintained at the University of Pittsburgh according the standards of the Association for Assessment and Accreditation of Laboratory Animal Care (AAALAC), and experiments were approved by the University of Pittsburgh Institutional Animal Care and Use Committee (IACUC). These studies were covered by the following IACUC protocol: 13011370. The animals were fed and housed according to regulations set forth by the *Guide for the Care and Use of Laboratory Animals* and the Animal Welfare Act [108]. All RMs included in this study were socially housed (paired) indoors in stainless steel cages, had 12/12 light cycle, were fed twice daily and water was provided *ad libitum*. A variety of environmental enrichment strategies were employed including housing of animals in pairs, providing toys to be manipulated, and playing entertainment videos in the animal rooms. Furthermore, the animals were observed twice daily and any signs of disease or discomfort were reported to the veterinary staff for evaluation. For sample collection, animals were anesthetized with 10mg/kg ketamine HCI (Park-Davis, Morris Plains, NJ, USA) or 0.7 mg/kg tiletamine (HCI) and zolazepan (Telazol, Fort Dodge Animal Health, Fort Dodge, IA) injected intramuscularly. At the end of the study, the animals were sacrificed by intravenous administration of barbituates.

### Animals, infections, and treatments

Ten Indian RMs were included in the study. They were infected with plasma equivalent to 300 tissue culture infectious doses (TCID50) of SIVsmmFTq [76,109] transmitted-founder infectious molecular clone. Clone derivation and preparation was similar to that reported by our group previously [77]. None of the RMs included in this study harbored MHC genotypes associated with control of SIV virus replication (i.e. A*01, B*08, or B*17). Further, RMs were selected to be either homozygous or heterozygous for the TRP allele of Trim5α [110] as the infectious molecular clone was constructed to bypass TFP restriction [110].

Sixty days post-SIVsmmFTq infection, after the resolution of acute infection and establishment of the chronic viral setpoint, ART was initiated in four RMs. ART consisted of the reverse transcriptase inhibitors (*R*)-9-(2-phosphonylmethoxypropyl) adenine (PMPA; tenofovir; 20mg/kg; gift from Gilead Biosciences) and β-2’, 3’-dideoxy-3’-thia-5-fluorocytindine (FTC; emtricitabine; 50mg/kg; gift from Gilead Biosciences) by once-daily subcutaneous injection, the integrase inhibitor L-870812 (20mg/kg; gift from Merck) *b*.*i*.*d*. for nine months. PMPA and FTC were administered subcutaneously, while L-870812 was administered orally. During treatment, after all the RMs controlled viral replication, one animal was euthanized due to an unrelated clinical condition (complications of anesthesia).

After 9 months of ART, prior to treatment cessation, RMs were treated with the LRA RMD (Istodax, Celgene Corporation, Summit, NJ) at a dose of 7 mg/m^2^ in a slow perfusion over four hours. After cessation of ART, the RM controllers received three additional doses of RMD in similar conditions every 35–50 days.

Finally, 42 days after the last RMD administration, RMs received the CD8^+^ cell-depleting monoclonal antibody M-T807R1 (NIH Nonhuman Primate Reagent Resource, Boston, MA) at a dose of 50mg/kg. Animals were closely clinically monitored for physical and physiological changes during all stages of the study.

### Sampling and sample processing

Blood was collected from all RMs as follows: three times prior to infection (-30, -15 and 0 dpi), biweekly for the first two weeks (4, 7, 10, and 14 dpi) and weekly thereafter. This sampling schedule was designed to monitor viral replication during the acute infection and establishment of the viral set point, at which time point ART was to begin after 5 consecutive similar PVL measurements. During ART treatment, a weekly sampling was designed to monitor for viral blips. Upon cessation of ART, blood was sampled every 3 days to monitor virus rebound.

After RMD and anti-CD8 mAb administration, the schedule of blood collection was as follows: 0, 4 and 6 hours post-treatment, followed by 1, 2, 5, 12, 14, 21, 28, 35 dpt.

Within one hour after blood collection, plasma was harvested and peripheral blood mononuclear cells (PBMCs) were separated from the blood using lymphocyte separation media (LSM, MPBio, Solon, OH).

Blood chemistries and complete blood counts (CBCs) were obtained from Marshfield Laboratories (Cleveland, OH) from serum and whole blood, respectively.

### Plasma viral load quantification

We monitored the levels of viral replication to assess treatment efficacy as well as the impact of RMD administration on the reservoir virus. Most samples were subject to a quantitative reverse-transcription PCR, as described previously [111]. For samples that achieved <50 copies/ml, a single copy assay (SCA) was performed, as described [83,112].

Large volumes of plasma (5–8 ml) were pooled and virus pelleted by ultracentrifugation at 170,000 x *g* for 30 min in a Sorvall T1270 rotor. To compensate for increased amounts of nonvirus materials in the plasma that can potentially interfere with accurate quantitation, a known amount of RCAS [83] was added to each sample prior to centrifugation. This serves as an internal control to monitor the overall efficiency of the assay. RNA was isolated as follows: virus pellets were suspended in 100 μl proteinase K for lysing and digestion; 400 μl of GuSCN/glycogen (glycogen acting as a carrier) was added and followed by 500 μl of isopropanol to precipitate RNA. The RNA samples were then resuspended in 65 μl Tris-HCl, pH 8.0, DTT, RNasin mixture. cDNA was first prepared in triplicate reaction mixtures for each RNA samples in a 96-well PCR plate. SIVsmmFTq gag standard (diluted to 1 copy/ml), two 7500 copy RCAS aliquots and corresponding water (negative control) were added the plate. Reaction mixtures contained 10 μl RNA and 12 μl cocktail [reverse transcriptase plus buffer, Superscript III First-strand synthesis Supermix for qRT-PCR kit (Invitrogen)]. cDNA was then synthesized under the following thermal conditions: 25°C for 10 min, 50°C for 50 min, 85°C for 5 min, and 4°C hold. Following reverse transcription, either SIVsmmFTq primers/probe for Gag (200 nM and 100 nM, respectively) or RCAS primers/probe and 30 μl Taqman Gene Expression Master (Applied Biosystems, Foster City, CA) were added to their respective wells. The primer and probe sequences are as follows: SIVFTqF: 5’-AAG TCC AAG AAC ACT GAA TGC ATG-3’; SIVFTqR: 5’-TAT AAT TTG CAT GGC TGC CTG ATG-3; SIVFTqProbe: 5’-/56-FAM/AGC GGA GGT/ZEN/AGT GCC AGG ATT CCA GGC/3IABkFQ/-3’; RCASF: 5’-GTC AAT AGA GAG AGG GAT GGA CAA A-3’; RCASR: 5’-TCC ACA AGT GTA GCA GAG CCC-3’; RCASProbe: 5’-/56-FAM/TGG GTC GGG/ZEN/TGG TCG TGC C/3IABkFQ/-3’. Real-time PCR and data assimilation were performed utilizing an ABI 7900 HT real-time machine under the following thermal profile: 95°C for 10min to activate the polymerase, followed by 50 cycles of 95°C for 15 seconds, 60°C for 1 min.

### Flow cytometry

To monitor the impact of ART on major immune cell populations with emphasis on CD4^+^ T cell restoration and immune activation, the immune cells were immunophenotyped by flow cytometry. First, a two-step TruCount technique was used to enumerate CD4^+^ and CD8^+^ T cells in blood, as previously described [113]. The number of CD45^+^ cells was quantified using 50 μl of whole blood stained with antibodies in TruCount tubes (BD Biosciences) that contained a defined number of fluorescent beads to provide internal calibration. The numbers of CD4^+^ and CD8^+^ T cells were then calculated based on the ratio of CD4^+^ and CD8^+^ T cells to CD45^+^ cells in whole blood at the same time point. Whole peripheral blood was stained with fluorescently-labeled antibodies (all antibodies from BD Bioscience, San Jose, CA, USA unless otherwise noted), CD4 (APC), HLA-DR (PE-Cy7), CD45 (PerCP), CD25 (PE), CD69 (APC-Cy7), CD20 (APC-H7), CD8 (PE-Texas Red) (Invitrogen) and CD38 (FITC) (Stemcell). For intracellular staining, cells were fixed, permeabilized and stained for Ki-67 (PE). Flow cytometry acquisitions were performed on an LSR II flow cytometer (BD Biosciences) and flow data were analyzed with FlowJo software (Treestar, Ashland, OR, USA).

### Cell sorting and cell-associated DNA quantification

To monitor the dynamics of changes in the virus reservoir due to RMD administration, frozen PBMCs were thawed and CD4^+^ total memory (effector and central memory) cells were sorted using magnetic bead kits and an AutoMACs Pro Separator (Miltenyi Biotec Cambridge, MA). Briefly, CD4^+^ cells were sorted by staining the PBMCs with the NHP CD4^+^ T cell isolation kit (Miltenyi Biotech). The sorted cells were then stained with CD95 (PE) (BD Bioscience, San Jose, CA), and then with anti-PE microbeads (Miltenyi Biotech) for the sorting of total memory cells. Sorted cells were pelleted and dry frozen for DNA extraction. After each sort, we removed 10^5^ cells for purity check.

The purity checks were performed as follows: cells from the first sort were stained with the following antibodies: CD3 (V450), CD8 (PE-C594), CD4 (PE), CD14 (FITC), CD20 (APC-H7), CD11c (APC), CD123 (Pe-Cy7), HLA-DR (PerCP). Cells from the second sort were stained with the following antibodies: CD3 (V450), CD4 (APC), CD95 (PE), CD28 (PE-Cy7). Stained cells were analyzed on a LSR II flow cytometer and flow data was analyzed using with FlowJo software. Purity checks confirmed that the purity of the sorted populations was higher than 90%.

Total DNA was extracted from the cell pellets using Qiagen DNeasy blood and tissue kit (Qiagen, Valencia, CA). Extracted DNA was then subjected to quantification using the same assays for the plasma vRNA quantification, but omitting the reverse transcription step. Simultaneous quantification of CCR5 was done to normalize sample variability and allow accurate quantification of cell equivalents. The CCR5 primer and probe sequences were: RMCCR5F: 5’- CCA GAA GAG CTG CGA CAT CC—3’; RMCCR5R: 3’- GTT AAG GCT TTT ACT CAT CTC AGA AGC TAA C—3’; RMCCR5Probe: 5’- /56-FAM/TTC CCC TAC/ZEN/AAG AAA CTC TCC CCG GTA AGT A/3IABkFQ—3’. Primers and probes were ordered from Integrated DNA Technologies (Integrated DNA Technologies (IDT), Coralville, IA), and the Taqman Gene Expression mix was from Applied Biosystems (Applied Biosystems, Foster City, CA). The detection limit of the viral DNA quantification assay was 30 copies/10^6^ cells.

### Histone acetylation assay

Treatment efficacy was determined by measuring the levels of histone acetylation in PBMCs using a flow cytometric assay, on samples collected prior to RMD administration and then at 6 hours, 1, 3, 5 dpt. Approximately 2 x 10^6^ freshly isolated PBMCs were surface immunophenotyped for 20 min at room temperature in the dark by using the following flow panel: CD69-brilliant violet (BV) 421 (FN50; Biolegend), CD4-V450 (L200), CD14-BV570 (M5E2; Biolegend), CD8-PE (SK1), CD28-ECD (CD28.2; Beckman Coulter), CD95-PE-Cy5 (DX2), PD-1-PE-Cy7 (EH12.2h7; Biolegend) and CD3-APC-Cy7 (SP34-2). Cells were immediately treated with PhosFlow lyse/fix buffer and incubated for 30 min at 37°C, washed twice, permeabilized with 0.4% Triton X-100 buffer (Sigma) for 10 min at room temperature in the dark, and washed again. Permeabilized cells were then stained intracellularly for 30 min at 4°C in the dark with the following antibodies: acetylated histone (recognizes several residues on histones H3 and H4; 3HHH4-2C2; Active Motif) and Ki-67-Alexa Fluor 647 (B56). Prior to use, the acetylated histone antibody was FITC labeled by using a Zenon reagent kit (Invitrogen), according to manufacturer’s instructions. After washing, stabilizing fixative was added, and approximately 200,000 CD3^+^ T cells were acquired for each sample by using a BD LSR-II flow cytometer. Population gating was performed using corresponding fluorescence minus one (FMO) and untreated negative control samples.

### ICS assay

Frozen PBMC samples were thawed, counted and treated with the following antibody cocktail: CD107α (BD), CD28 (BD), and CD49d (BD) in R10 media. The cells were stimulated by four conditions: SIVmac239 Env peptide pool, SIVmac239 Gag peptide pool, positive control staphylococcal enterotoxin B (SEB), and negative control DMSO. SIVmac239 Env and Gag peptide pools were obtained through the AIDS reagent program, Division of AIDS, NIAID, NIH. The samples were incubated for 2 hours at 37°C. Cells were then treated with Brefeldin A (Sigma) and monensin (Sigma) for 4 hours at 37°C. Cells were then stained with Blue LIVE/DEAD (Invitrogen), CD4 (APC), CD8α/β (PE-Texas Red) and CD3 (V450) for surface and TNFα (AF700), IFNγ (FITC), IL-2 (PE), and MIP-1β (PE-Cy7) for intracellular. Samples were run on a LSR-II flow cytometer and analyzed with FlowJo software.

### LDH levels in plasma

To investigate if leukopenia observed after RMD administration is due to a real destruction of leukocytes following RMD administration, LDH levels in plasma were quantified by ELISA according to the manufacturer’s protocol (NeoBioLab, Cambridge, MA, USA). Results were expressed in ng/ml plasma and the ranges of detection were 5–100 ng/ml. In healthy human individuals, the LDH levels range from 5.6 to 226 ng/mL.

### Statistical analysis

Levels of LDH were compared between D0 and mean peak using paired t-test. Differences in the levels of leukocytes and immune activation markers were determined using Mann-Whitney U test. GraphPad Prism 6 (Graphpad software) was used for all statistical analysis except for mixed-effects models, which were determined using R.

### Mathematical modeling

To analyze the impact of RMD on activating cells into viral production we used a simple model, similar to a previously published analysis [114]. We assume that before each cycle of RMD after ART interruption, the PVL is in approximate steady state, as indicated by the data. Virus is produced at a constant rate P and is removed at rate c per virus, such that the change in viral load is described by $\frac{dV}{dt}=P-cV$. Before RMD, the PVL is approximately constant, dV/dt = 0, and thus P_0_ = cV_0_, where we use the subscript to indicate time 0 of each RMD cycle. Note that at each cycle, the initial viral load (*V*_*0*_) can be different. At each RMD dose, we assume that the production of virus is increased by the recruitment of latent cells into productive infection. We model the early increase of viral production by $\frac{dV}{dt}=(P_{0}+P_{R})-cV$. This is only valid before new cycles of infection ensue, because then viral production (*P*_0_+*P*_R_) is no longer constant. This model can be solved to yield $V(t)=\frac{P_{0}}{c}+\frac{P_{R}}{c}(1-e^{-ct})$. By taking the log_10_ of *V*(*t*) and expanding the result in a Taylor series to first order, we conclude that early on the PVL changes as

$${\text{log}}_{10}(V(t))\approx \frac{\text{ln}(P_{0}/c)}{\text{ln}(10)}+\frac{cP_{R}}{P_{0}\text{ln}(10)}t=\frac{\text{ln}(V_{0})}{\text{ln}(10)}+\frac{P_{R}}{V_{0}\text{ln}(10)}t,$$

where “ln” represents the natural logarithm, and we used that *P*_0_ = *cV*_0_ to obtain the last expression. These expressions show that the log_10_ of PVL should grow approximately linearly in time early on after RMD treatment, with slope given by the term multiplying time, *t*. Below we will use both of the above expressions.

We fitted a linear mixed effects model to the log_10_ of PVL between 0 dpt of each cycle of romidepsin treatment and the maximum PVL in each case, using the nlme package of R [115]. We used “Time” and “Cycle” of RMD treatment as fixed factors and RMs as a random factor. We check homogeneity of variance (plot of residuals) and normality of error (Normal qq-plot). We found that Cycle was a significant factor, but there was no interaction between Cycle and Time. We also found that the slope of increase over time was not significantly different among the three subjects. Thus, our final mixed-effects linear model that best fitted the initial increase in log_10_ PVL had a different intercept (*i*.*e*., estimated initial PVL, *V*_0_) for each macaque and Cycle, but the same slope (S) of increase in all cases, and can be represented by

$${\text{log}}_{10}(V_{M}^{c})=V_{0}{}_{,M}^{c}+St,$$

where c = 1, 2, 3 represents the RMD cycle number after ART cessation and M corresponds to each RM. The fitting estimated *V* ^c^_0,M_ and *S* in each case.

Putting the two approaches together, the linear mixed-effects model estimates with the dynamical model expressions, we see that *S* = *P*_*R*_/(*V*_0_ln(10)). Thus, we can estimate P_R_, which is the increase in viral production per ml and per unit time due to RMD. We multiply this number by the estimated total blood volume of about 500 ml to obtain the total production. In addition, we see also that *S* = *cP*_*R*_/(*P*_0_ln(10)), allowing estimation of *P*_*R*_/*P*_0_, which is the relative increase in production of virus over baseline due to treatment. In this last case, we need to know *c* for which we use a range of c≈20 day^-1^ [114] to c≈100 day^-1^ [116].

## Supporting Information

S1 Fig Assessment of the RMD toxicity in SIVsmmFTq-infected post-treatment controller RMs. Testing a comprehensive chemistry panel did not reveal any significant increase in the levels of different metabolites (suggestive of cell toxicity) between the samples collected prior to and after RMD administration.
(PDF)

S2 Fig Assessment of the impact of RMD on major blood cells. Differential cell blood counts (CBCs) were performed before and after RMD administration to investigate the effect of the drug on major cell components.
(PDF)

S3 Fig Average plasma LDH levels during various rounds of RMD therapy. The LDH levels, measured using a quantitative ELISA and are expressed in ng/ml, did not significantly increase after administration of RMD. The lines represent the average LDH levels of the three RMs receiving RMD and the bars represent the sem. The RMD treatments are color-coded.
(PDF)

S4 Fig The boost of viral replication observed in SIVsmmFTq-infected post-treatment controller RMs was due to target cell reactivation by RMD. Plotting of the levels of different immune activation makers, i.e., (a) CD69; (b) HLA-DR and CD38; and (c) CD25 showed that the increase in immune activation always precedes the virus rebound in all treated animals. Data presented are representative for all animals and all markers. Times of the RMD administration are illustrated with black arrows.
(PDF)

S5 Fig RMD administration did not significantly impact CTL responses or functionality in SIVsmmFTq post-treatment controller RM135. Serial monitoring of CTL polyfunctionality after two rounds of RMD administration was achieved by stimulating PBMCs with either (a) Gag or (b) Env SIVmac239 peptide pools followed by intracellular cytokine staining. Cytokines tested for include: TNF-α (T); IL-2 (2); IFN-γ (I); CD107α (7); and MIP-1β (M). Data are representative of all RMs. Absolute numbers of CD4^+^/CD8^+^ T cells/ml for each timepoint are present beneath their respective pie graph. The pie charts depict functionality based on the combination of cytokines expressed, as illustrated in figure legends. The color scheme represents the number of cytokines produced by the CTLs and the proportion of each is illustrated as a color-coded ring surrounding each pie chart to facilitate assessment of polyfunctionality.
(PDF)

S6 Fig RMD administration did significantly impact CTL responses or functionality in SIVsmmFTq post-treatment controller RM140. Serial monitoring of CTL polyfunctionality after two rounds of RMD administration was achieved by stimulating PBMCs with either (a) Gag or (b) Env SIVmac239 peptide pools followed by intracellular cytokine staining. Cytokines tested for include: TNF-α (T); IL-2 (2); IFN-γ (I); CD107α (7); and MIP-1β (M). Data are representative of all RMs. Absolute numbers of CD4^+^/CD8^+^ T cells/ml for each timepoint are present beneath their respective pie graph. The pie charts depict functionality based on the combination of cytokines expressed, as illustrated in figure legends. The color scheme represents the number of cytokines produced by the CTLs and the proportion of each is illustrated as a color-coded ring surrounding each pie chart to facilitate assessment of polyfunctionality.
(PDF)

S7 Fig After CD8^+^ cell depletion, the boost of viral replication observed in SIVsmmFTq-infected post-treatment controller RMs was due to ablation of the immune control. Plotting of the levels of different immune activation makers, i.e., CD69; HLA-DR and CD38; CD25; and Ki-67 showed that the increase in immune activation always occurred after the virus rebound in all treated animals. Data presented are representative for all the animals and all the markers. Times of the M-T807R1 administration are illustrated with red arrows.
(PDF)
