# Supplementary figures and images for: Multi-dose Romidepsin Reactivates Replication Competent SIV in Post-antiretroviral Rhesus Macaque Controllers
Source: PLoS Pathog. 2016 Sep 15;12(9):e1005879. doi: 10.1371/journal.ppat.1005879 (PMC5025140; doi:10.1371/journal.ppat.1005879)

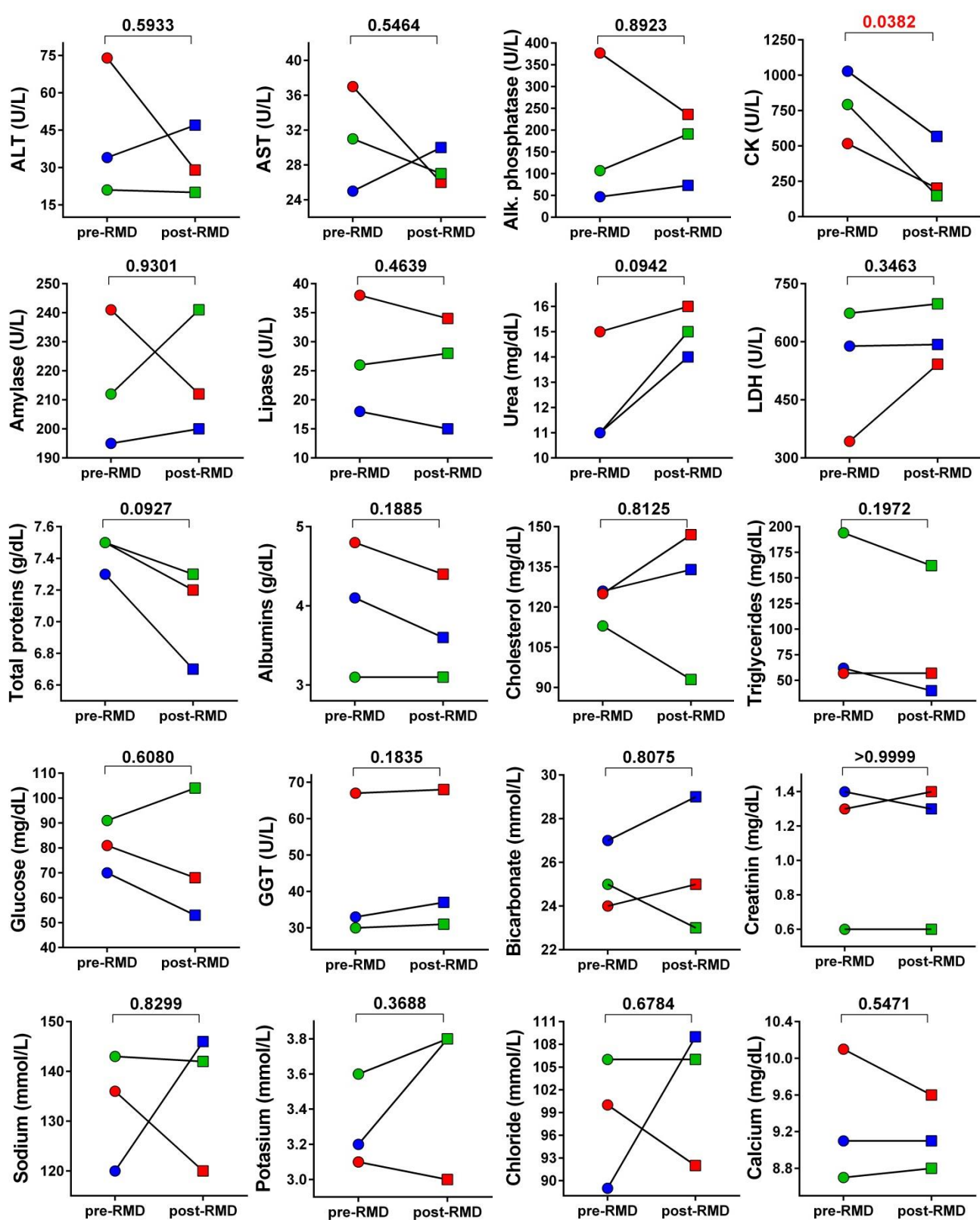

Supplement: S1 Fig — Testing a comprehensive chemistry panel did not reveal any significant increase in the levels of different metabolites (suggestive of cell toxicity) between the samples collected prior to and after RMD administration. (PDF) [file ppat.1005879.s001.pdf]

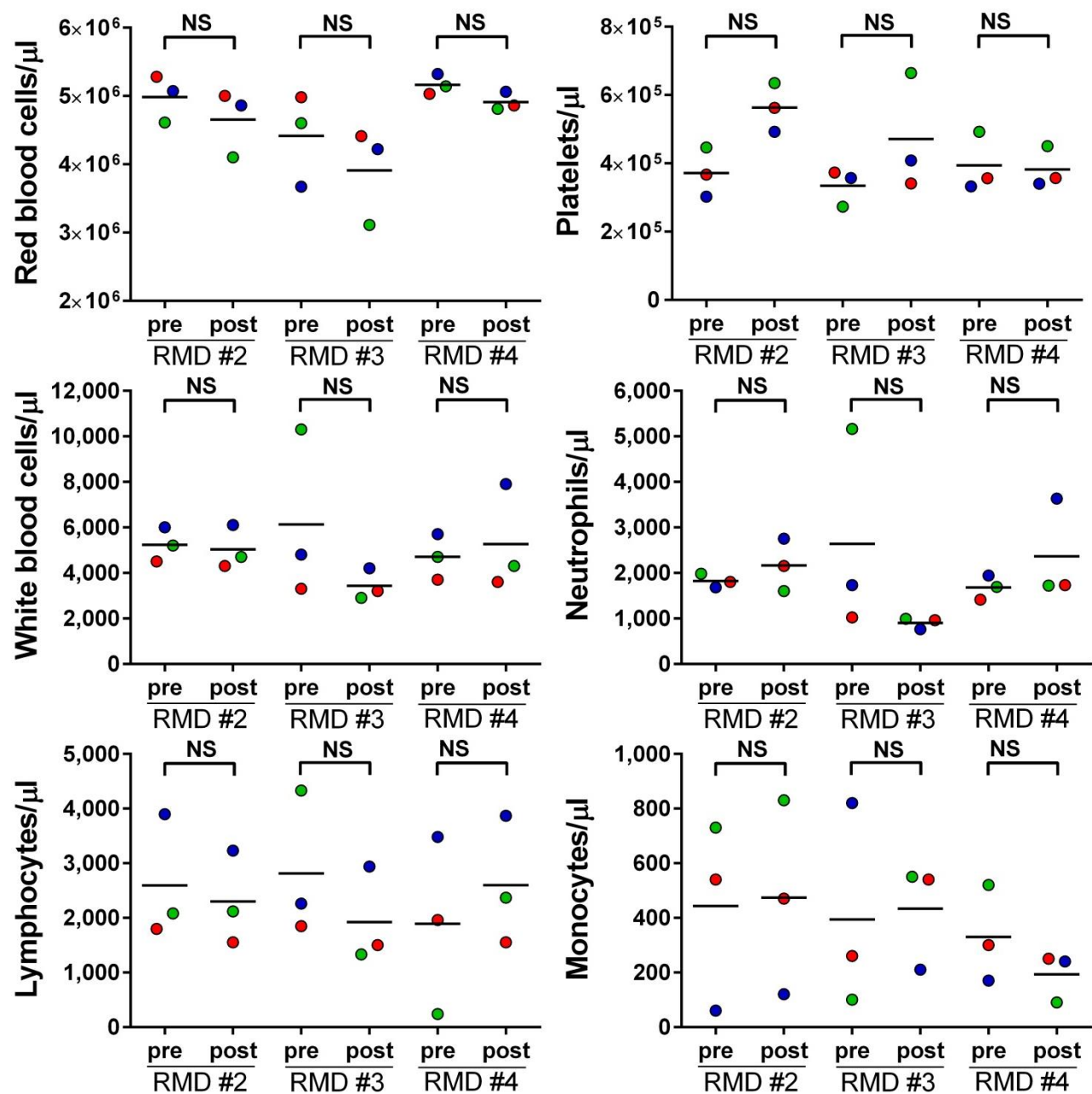

Supplement: S2 Fig — Differential cell blood counts (CBCs) were performed before and after RMD administration to investigate the effect of the drug on major cell components. (PDF) [file ppat.1005879.s002.pdf]

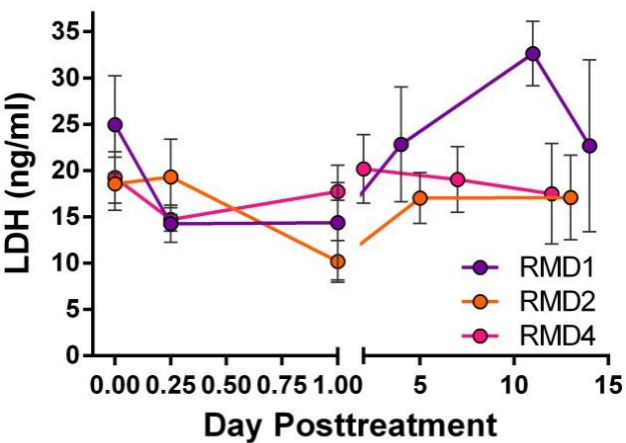

Policicchio et al., Figure S3

Supplement: S3 Fig — The LDH levels, measured using a quantitative ELISA and are expressed in ng/ml, did not significantly increase after administration of RMD. The lines represent the average LDH levels of the three RMs receiving RMD and the bars represent the sem. The RMD treatments are color-coded. (PDF) [file ppat.1005879.s003.pdf]

### a. RM135

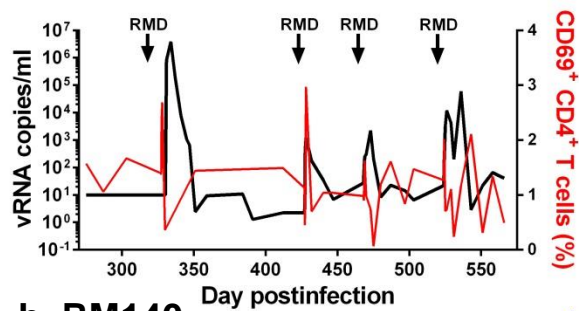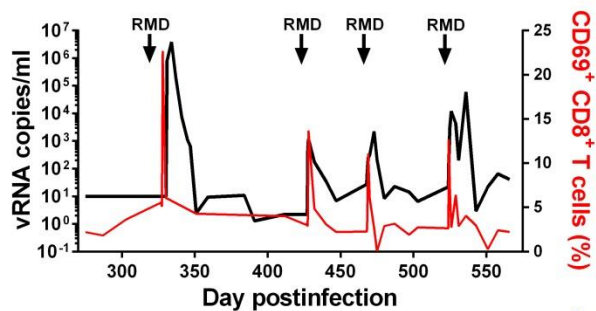

### b. RM140

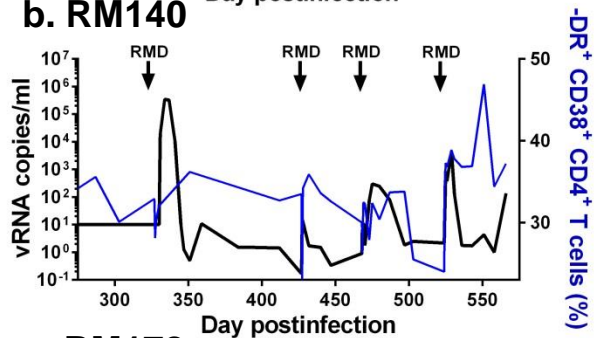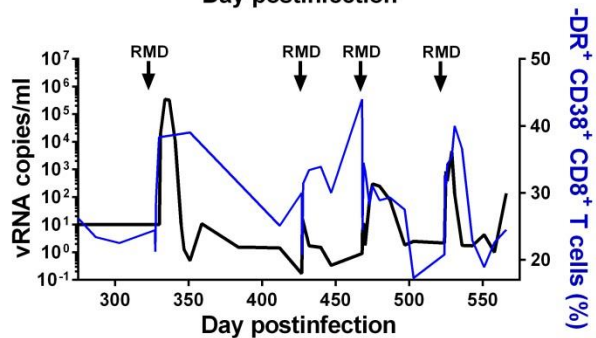

### c. RM178

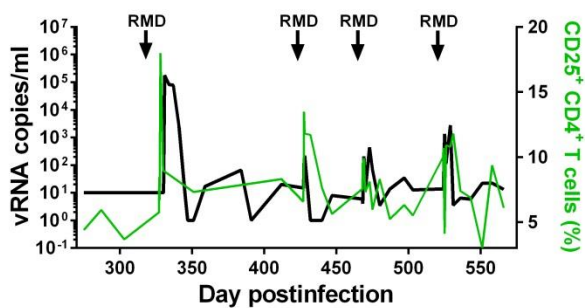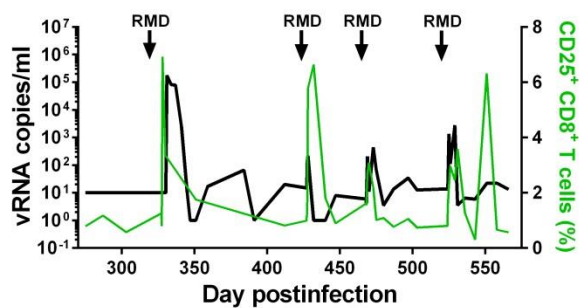

Supplement: S4 Fig — Plotting of the levels of different immune activation makers, i.e., (a) CD69; (b) HLA-DR and CD38; and (c) CD25 showed that the increase in immune activation always precedes the virus rebound in all treated animals. Data presented are representative for all animals and all markers. Times of the RMD administration are illustrated with black arrows. (PDF) [file ppat.1005879.s004.pdf]

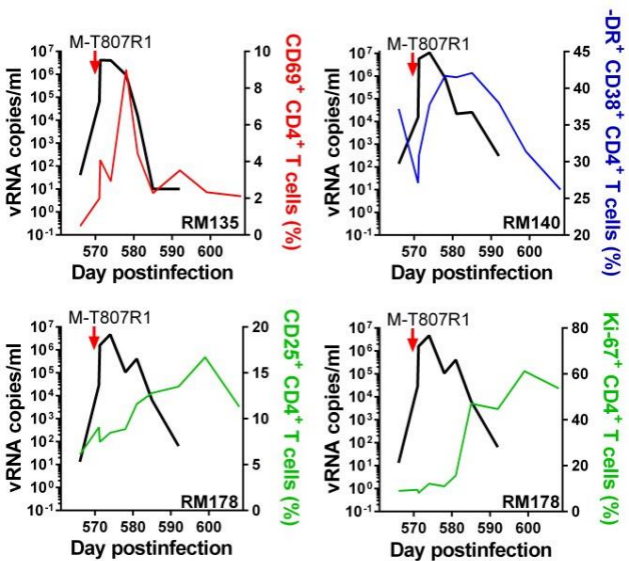

Supplement: S7 Fig — Plotting of the levels of different immune activation makers, i.e., CD69; HLA-DR and CD38; CD25; and Ki-67 showed that the increase in immune activation always occurred after the virus rebound in all treated animals. Data presented are representative for all the animals and all the markers. Times of the M-T807R1 administration are illustrated with red arrows. (PDF) [file ppat.1005879.s007.pdf]
