# Supplementary material for: Multi-dose Romidepsin Reactivates Replication Competent SIV in Post-antiretroviral Rhesus Macaque Controllers
Source: PLoS Pathog. 2016 Sep 15;12(9):e1005879. doi: 10.1371/journal.ppat.1005879 (PMC5025140; doi:10.1371/journal.ppat.1005879)
Supplement: S5 Fig — Serial monitoring of CTL polyfunctionality after two rounds of RMD administration was achieved by stimulating PBMCs with either (a) Gag or (b) Env SIVmac239 peptide pools followed by intracellular cytokine staining. Cytokines tested for include: TNF-α (T); IL-2 (2); IFN-γ (I); CD107α (7); and MIP-1β (M). Data are representative of all RMs. Absolute numbers of CD4+/CD8+ T cells/ml for each timepoint are present beneath their respective pie graph. The pie charts depict functionality based on the combination of cytokines expressed, as illustrated in figure legends. The color scheme represents the number of cytokines produced by the CTLs and the proportion of each is illustrated as a color-coded ring surrounding each pie chart to facilitate assessment of polyfunctionality. (PDF) [file ppat.1005879.s005.pdf]

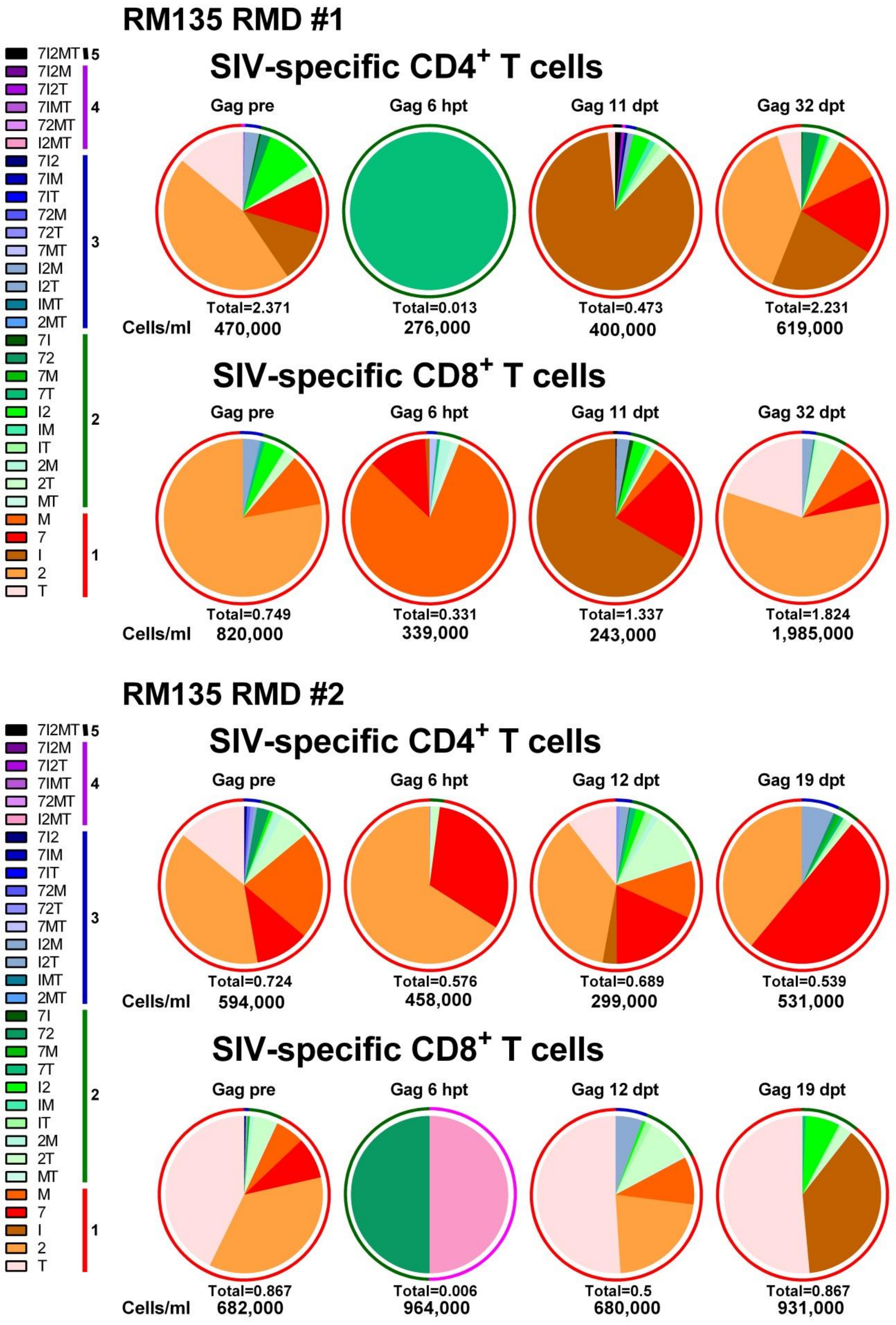

Policicchio et al., Figure S5a

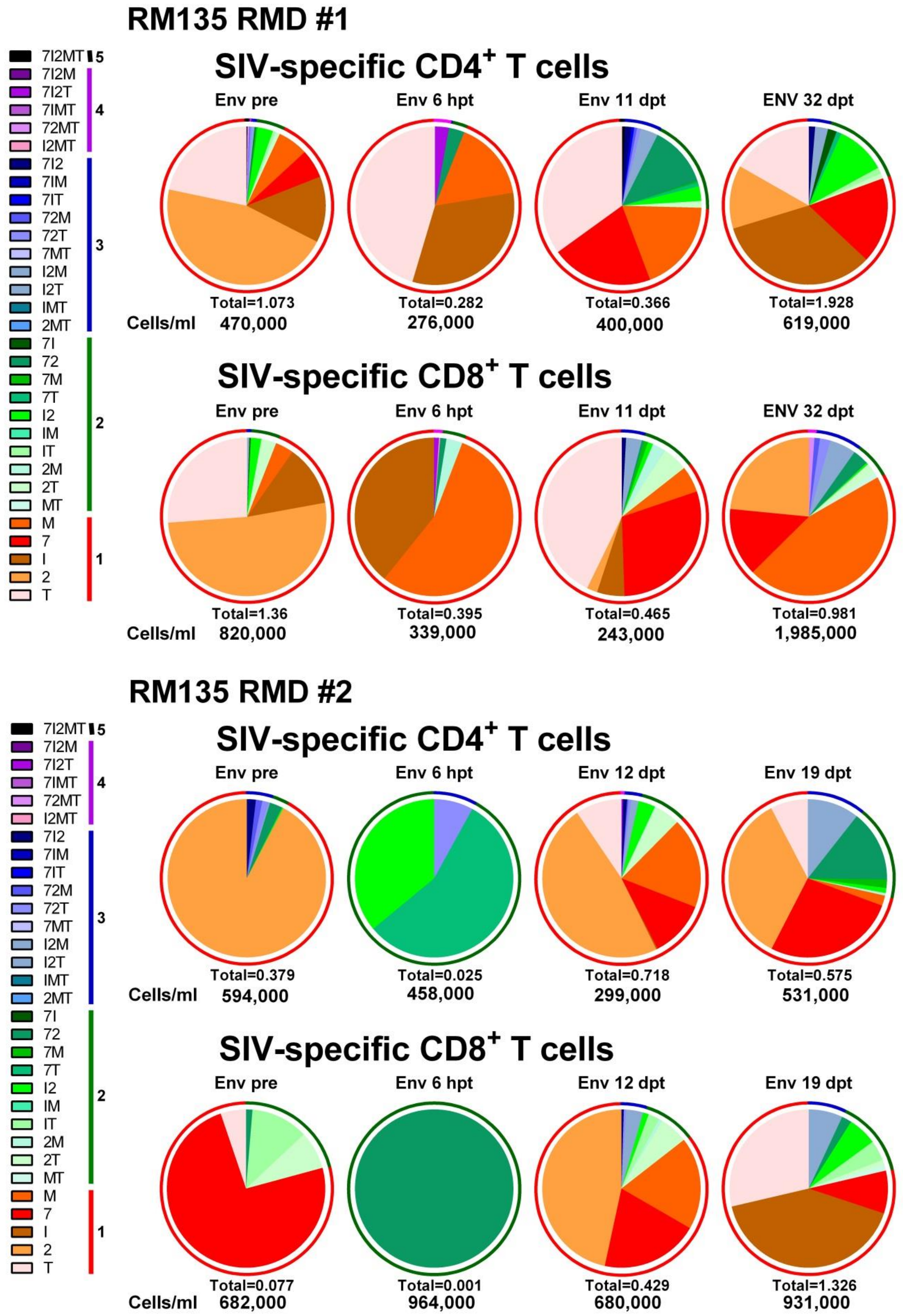

Policicchio et al., Figure S5b
